# UCPVax, a CD4 helper peptide vaccine, induces polyfunctional Th1 cells, antibody response, and epitope spreading to improve antitumor immunity

**DOI:** 10.1016/j.xcrm.2025.102196

**Published:** 2025-06-20

**Authors:** Caroline Laheurte, Laura Boullerot, Babacar Ndao, Marine Malfroy, Lise Queiroz, Phillippe Guillaume, Romain Loyon, Evan Seffar, Eleonore Gravelin, Adeline Renaudin, Marion Jacquin, Aurélia Meurisse, Dewi Vernerey, François Ghiringhelli, Yann Godet, Raphael Genolet, Camilla Jandus, Christophe Borg, Olivier Adotévi

**Affiliations:** 1 Université Marie et Louis Pasteur, EFS, INSERM, UMR RIGHT, 25000 Besançon, France; 2 Ludwig Institute for Cancer Research, Lausanne Branch, University of Lausanne and Lausanne University Hospital, Lausanne, Switzerland; 3 Computational Oncology Service, Department of Epidemiology and Biostatistics, Memorial Sloan Kettering Cancer Center, New York, NY 10065, USA; 4 Clinical Investigational Center, CIC-1431, Centre Hospitalier Universitaire de Besançon, Besançon, France; 5 Methodology and Quality of Life in Oncology Unit, University Hospital of Besançon, Besançon, France; 6 Department of Medical Oncology, Centre Georges-François Leclerc, Dijon, France; 7 Department of Oncology UNIL CHUV, University Hospital of Lausanne, Lausanne, Switzerland; 8 Department of Pathology and Immunology, Faculty of Medicine, University of Geneva, Geneva, Switzerland; 9 Geneva Center for Inflammation Research, Geneva, Switzerland; 10 Department of Medical Oncology, University Hospital of Besançon, 25000 Besançon, France

**Keywords:** CD4^+^ T cells, helper peptide, cancer vaccine, telomerase, polyfunctional T cell, antibody response, epitope spreading, lung cancer

## Abstract

The induction of an antitumor CD4^+^ T helper response is essential for the efficacy of therapeutic cancer vaccines. However, few vaccines are specifically designed to target CD4^+^ T cells in human cancers. Here, we characterize the immune mechanisms of UCPVax, a helper peptide vaccine derived from telomerase. *Ex vivo* immune profiling of peripheral blood from 60 patients with advanced lung cancer reveals that UCPVax selectively activates CD4^+^ T cells *in vivo* across a broad HLA-DR restriction. The vaccine elicits a synergistic immune triad, including cytokine polyfunctional CD4^+^ Th1 cells, epitope spreading, and antibody response, contributing to effective tumor control. Single-cell analysis further demonstrates that UCPVax drives CD4^+^ T cells toward effector memory and cytolytic differentiation. Thus, vaccine-induced CD4^+^ T cells trigger broad and durable antitumor immunity. These findings highlight UCPVax as an off-the-shelf helper platform to enhance therapeutic cancer vaccine efficacy. This study was registered at ClinicalTrials.gov: NCT02818426.

## Highlights

• UCPVax vaccination stimulates CD4^+^ T cells across a broad MHC class II context
• UCPVax drives CD4^+^ T cells toward polyfunctional and cytolytic Th1 polarization
• Induction of strong epitope spreading and antibody response after UCPVax vaccination
• These UCPVax-induced immune responses contribute to tumor control

## Graphical abstract

**Figure:**
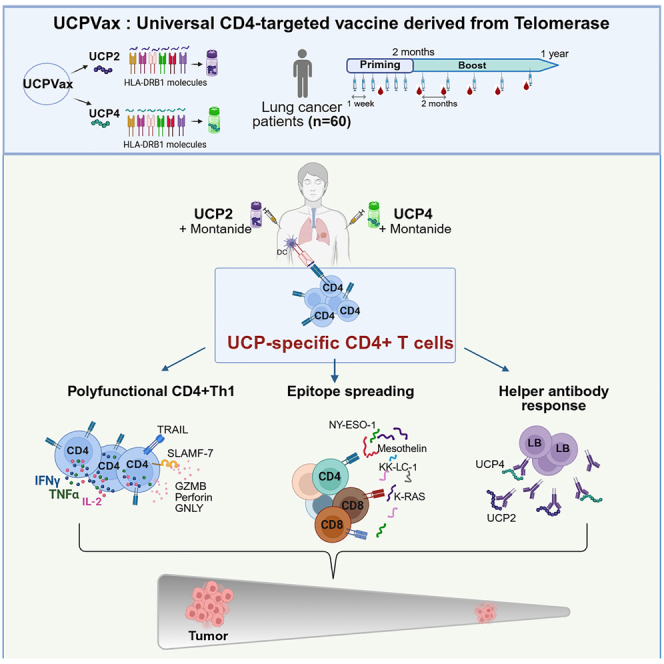

## Teaser

CD4^+^ T cell stimulation is critical for cancer vaccine efficacy. Laheurte et al. describe the properties of UCPVax, a CD4 helper cancer vaccine derived from telomerase that triggers polyfunctional Th1 cells with cytolytic properties, generates robust antibody responses, and promotes strong epitope spreading—factors that contribute to optimal tumor control.

## Introduction

Cancer vaccines have garnered significant interest as supportive, combinatory, or personalized therapies for patients with cancer. Although earlier therapeutic cancer vaccines showed limited success, a new era is emerging by addressing the factors behind past failures.^1^^,^^2^ One key factor contributing to the limited efficacy of previous anticancer vaccine trials is the insufficient stimulation of tumor-reactive CD4^+^ T cells. Previous observations underscore the critical role of CD4^+^ T cell activation in enhancing the effectiveness of cancer vaccines.^1^^,^^2^^,^^3^ Additionally, recent evidence highlights the clinical benefits of neoantigen vaccines, particularly those that elicit tumor-reactive CD4^+^ T cell responses.^4^^,^^5^^,^^6^^,^^7^

Several aspects of CD4^+^ T cell biology suggest that this population can be effectively harnessed for cancer immunotherapy.^8^^,^^9^ Among the various CD4^+^ T cell subtypes that regulate immune responses, the CD4^+^ T helper 1 (Th1) subset, which produces interferon (IFN)γ, tumor necrosis factor alpha (TNF-α), and interleukin-2 (IL-2), plays a central antitumor role by orchestrating cell-mediated immunity against cancer.^10^^,^^11^ Th1 cells enhance the generation, function, and memory of cytotoxic CD8^+^ T cells.^10^^,^^12^^,^^13^^,^^14^^,^^15^ Importantly, tumor-reactive CD4^+^ T cells facilitate the entry of effector CD8^+^ T cells into the tumor microenvironment (TME).^13^^,^^16^ Additionally, CD4^+^ T cells provide support to B cells and other immune effectors, such as natural killer cells and type 1 macrophages.^8^^,^^17^ Beyond their helper functions, CD4^+^ T cells exhibit other antitumor properties. Through IFNγ secretion, they regulate vascular normalization within the TME, promoting hypoxia attenuation and immune activation.^18^^,^^19^ Furthermore, cytotoxic CD4^+^ T cell subsets can directly eliminate tumor cells, either in a major histocompatibility complex (MHC) class II-dependent or -independent manner.^20^^,^^21^^,^^22^ All these findings support the observation that a CD4^+^ Th1-associated immune signature in the TME is associated with positive prognostic markers in several human cancers.^23^^,^^24^ Despite this compelling evidence, few cancer vaccines are specifically designed to stimulate CD4^+^ Th1 cells.^25^ This paradox may stem from the inherent plasticity of CD4^+^ T cells,^26^^,^^27^ which presents a potential risk of inducing pro-tumoral immune responses, such as the differentiation of regulatory T cells (Tregs).^8^^,^^28^

A well-established approach to stimulating CD4^+^ T helper responses in cancer vaccines involves using xenogeneic or non-tumor-specific peptides, such as the synthetic helper peptide PADRE (derived from keyhole limpet hemocyanin [KLH]) and tetanus toxoid-derived helper peptides.^29^^,^^30^ However, these strategies provide non-tumor-specific immune activation, and their clinical benefit remains uncertain.^25^^,^^31^^,^^32^ Recent melanoma studies demonstrated that vaccines incorporating melanoma antigen-derived helper peptides induced durable and protective immune responses, significantly surpassing those observed with tetanus-derived helper peptides.^33^^,^^34^ Despite these promising results, the full spectrum of immune responses elicited by cancer vaccines targeting CD4^+^ helper T cells remains to be extensively explored in the clinic, particularly considering the plasticity of CD4^+^ T cell subsets.

To address this, we developed UCPVax, a CD4^+^ Th1-inducing cancer vaccine. UCPVax consists of two highly pan-HLA-DR (human leukocyte antigen)-binding peptides, termed UCPs (universal cancer peptides),^35^^,^^36^^,^^37^ derived from human telomerase reverse transcriptase (hTERT)—a key hallmark of cancer and a widely recognized tumor-associated antigen (TAA) for immunotherapy.^38^^,^^39^^,^^40^ UCPVax was recently evaluated in a phase 1/2 clinical trial in patients with advanced non-small cell lung cancer (NSCLC), demonstrating a favorable safety profile and preliminary signs of efficacy across all dose levels.^41^ In the present study, we investigate the antitumor immune mechanisms induced by UCPVax-mediated CD4^+^ T cell stimulation. Through an in-depth immune analysis of 60 previously vaccinated patients with advanced NSCLC, we reveal that UCPVax induces highly polyfunctional (polyF) CD4^+^ Th1 cells with cytolytic properties, generates robust UCP-specific antibody (Ab) responses, and promotes strong epitope spreading—factors that contribute to optimal tumor control.

## Results

### Patients, study design, and overall immunogenicity

**Figure 1:**
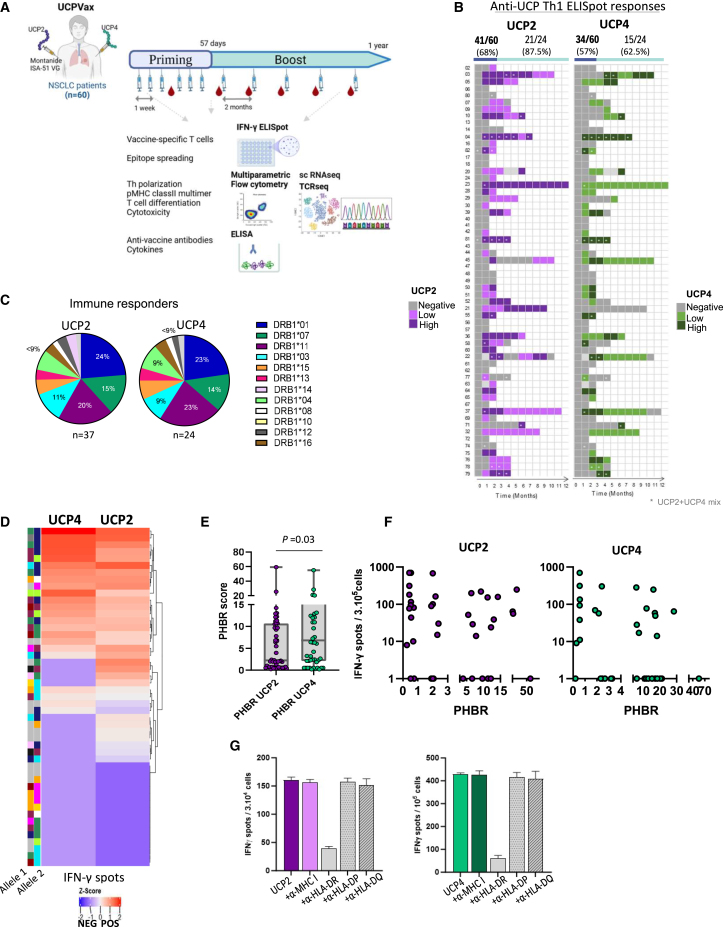
UCPVax induces hTERT-reactive CD4^+^ T cells in a broad range of HLA class II contexts (A) Treatment plan of UCPVax. UCPVax includes UCP2 and UCP4 pan-HLA-DR binding helper peptides derived from telomerase (hTERT) as previously described (Adotévi et al.^41^). Each helper peptide was emulsified in the adjuvant Montanide ISA-51 VG and injected subcutaneously. Patients received 6 weekly injections (priming) following by booster vaccinations for 8 weeks (boost) for a maximum of 12 months. Blood samples were collected for immune readouts as indicated (created with BioRender.com). (B) Heatmap showing distribution of vaccine-induced UCP2- and UCP4-specific CD4^+^ T cells over time (*n* = 60). The frequency of immune responders is shown at the top of the heatmap, intensity of response is shown at the bottom, and low and high responders were defined according to the median of IFNγ spots for each peptide (median spots: UCP2 = 84 and UCP4 = 63). The ∗ denotes patients evaluated with a mix of UCP2+UCP4. (C) Distribution of HLA-DR-B1 allele frequencies in patient responders to UCP2 (*n* = 37) and UCP4 (*n* = 24). (D) Heatmap representing HLA-DR-B1 allele expression in responders to UCP2 and UCP4 and in non-responders. (E) PHBR scores for UCP2 and UCP4. Boxplot represents median ± 1^st^ and 3^rd^ quartiles, Mann-Whitney test. (F) PHBR score according to the intensity of anti-UCP2 or UCP4 CD4^+^ T cell responses. (G) HLA-DR restriction. Histograms representing example of UCP2- (left) and UCP4- (right) specific responses with or without indicated MHC class I and II blocking antibodies measured by *ex vivo* ELISpot. Results represent mean of triplicates + SD. See also Figure S1 and Tables S1 and S2.

Sixty patients with refractory metastatic NSCLC were enrolled and received UCPVax, a vaccine composed of two pan-HLA-DR-binding Th1 epitopes derived from hTERT (UCP2 and UCP4), emulsified in the Montanide ISA51 adjuvant. The vaccine was administered subcutaneously at separate sites weekly for the first six doses (priming), followed by booster doses every 8 weeks for up to 1 year. An extensive *ex vivo* immune correlation study was conducted on peripheral blood samples (Figure 1A). Demographic and main baseline disease characteristics are summarized in Table S1. The ability of UCPVax to prime UCP-specific CD4^+^ T cells was first evaluated by direct *ex vivo* IFNγ ELISpot assays. After priming vaccinations, robust UCP2- and UCP4-specific CD4^+^ T cells were expanded in 41 (68%) and 34 (57%) out of the 60 evaluable patients, respectively. In patients who received booster vaccinations (*n* = 24), the longitudinal assessment showed that long-lasting specific responses were still detected *ex vivo* against UCP2 for 87.5% of patients (21/24) and against UCP4 for 62.5% (15/24) of patients (Figures 1B and S1A). The overall immunogenicity with a pooled UCP-specific CD4^+^ T cell response was 68% (Figures S1B and S1C). Since the UCP vaccine helper peptides were specifically designed to bind the most prevalent HLA-DR alleles, HLA genotyping was not required before vaccination.^35^^,^^37^ However, genotyping was performed in all patients to assess the impact of MHC class II restriction on CD4^+^ T cell induction by UCPVax. The distribution of HLA-DRB1 alleles in this cohort was consistent with that reported in the White population (Figure S1D). HLA-DR alleles were well balanced between immune responders to UCP2 and UCP4 (Figure 1C; Table S2). No obvious difference in HLA allele distribution was observed in immune non-responders to the vaccine (Figures 1D and S1E; Table S2). To assess the HLA-DR presentation of the vaccine peptides, we calculated the estimated HLA-DR PHBR (patient harmonic-mean best rank) score for UCP2 and UCP4 across all HLA-DRB1 genotypes.^42^ Results showed that the HLA-DR PHBR score of UCP2 was lower than that for UCP4 (Figure 1E), indicating a stronger HLA-DR binding affinity for UCP2, consistent with previous findings.^35^ However, no correlation was observed between the PHBR score and the magnitude of UCP-specific CD4^+^ T cell responses induced post-vaccination (Figure 1F; Table S2). Additionally, we found that UCP-specific CD4^+^ T cell activation was inhibited by an HLA-DR blocking Ab but not by anti-DP, anti-DQ, or anti-HLA class I blocking Abs, confirming that UCPVax effectively induces CD4^+^ T cell responses across a broad range of HLA-DR alleles (Figures 1G and S1F).

Next, we compared baseline characteristics between immune responders and non-responders to the vaccine. Among the main clinical factors considered, we observed a trend toward a longer duration of prior anti-PD-1 therapy in immune responders (Table 1). Additionally, baseline immune fitness parameters in peripheral blood revealed that non-responders exhibited higher neutrophil-to-lymphocyte ratios (NLRs) and elevated levels of inflammatory cytokines, such as IL-6 and IL-8 (Table 1).

**Table 1:** Baseline parameters according to immune response to UCPVax

	*n*	Immune non-responders	*n*	Immune responders	*p* value
Age, years, mean (SD)	19	67.4 (9.5)	41	67.1 (7.2)	0.90
Gender, no. (%)	19	–	41	–	–
Men	12	(63)	29	(71)	0.56
Women	7	(37)	12	(29)	–
Histology, no. (%)	19	–	41	–	0.79
Adenocarcinoma	11	(58)	24	(58)	–
Squamous cell	8	(42)	14	(34)	–
Others	–	0	3	(7)	–
KRAS mutations, no. (%)	12	–	25	–	0.27
WT	9	(75)	14	(56)	–
Mutated	3	(25)	11	(44)	–
PD-L1 expression, no. (%)	12	–	25	–	–
No	7	(58)	13	(52)	0.75
Yes	5	(42)	12	(48)	–
Duration previous anti-PD-(L)1, months, mean (SD)	19	4.6 (4.8)	41	8.5 (10.1)	0.06
Total lymphocyte count, mean (SD)	18	1,325.5 (1,040.4)	41	1,380.6 (574.7)	0.20
Neutrophil/lymphocyte ratio (NLR), mean (SD)	19	6.0 (3.6)	41	4.6 (3.2)	0.06
T CD4%, mean (SD)	18	50.0 (14.8)	41	51.3 (17.6)	0.77
T CD4 count, mean (SD)	18	743.9 (773.5)	41	719.4 (409.7)	0.38
Regulatory T cell (Tregs)%, mean (SD)	17	5.7 (2.1)	13	5.5 (2.2)	0.82
LDH, mean (SD)	17	299.5 (124)	39	270.6 (105)	0.43
Inflammatory cytokines, mean (SD)	12	–	30	–	–
IL-1β	–	0.17 (0.15)	–	0.56 (1.8)	0.88
IL-6	–	15.0 (16.3)	–	6.3 (7.3)	0.07
IL-8	–	25.6 (21.8)	–	16.4 (27.1)	0.03

No., number; SD, standard deviation.

### UCPVax vaccination elicits strong cytokine polyF CD4^+^ Th1 cells detectable *ex vivo*

**Figure 2:**
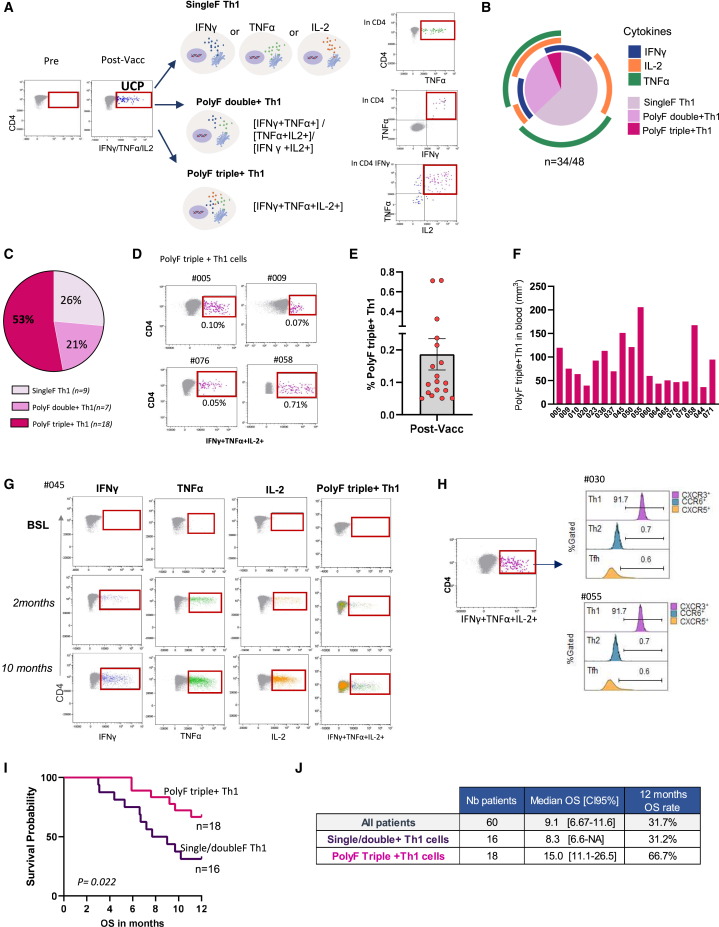
Expansion of cytokine polyfunctional triple CD4^+^ Th1 cells after UCPVax vaccination (A) Vaccine-induced UCP-specific CD4^+^ Th1 cells by *ex vivo* ICS assay in 48 patients. Left, schematic representation of UCPVax-expanded Th1 subset: mono-functional Th1, polyF double+ Th1, and polyF triple+ Th1 cells. Right, cytometry dot plots showing representative patients of each subset. (B) Nested pie chart showing the proportion (median) of each subset of UCP-specific Th1 cells (*n* = 48). (C) Pie chart showing the repartition of patients according to UCPVax-expanded Th1 cell subsets (*n* = 34). (D) Representative dot plots of patients with UCP-specific polyF triple+ Th1 cells. (E) Histograms showing rate of polyF triple+ Th1 cells in vaccinated patients (*n* = 18). Mean ± SD. (F) Count of polyF triple+ Th1 cells in the blood per mm^3^. (G) Representative examples of cytometry dot plots showing UCP-specific CD4^+^ T cells producing IFNγ, IL-2, and TNF-α at individual secretion and simultaneously along vaccination. (H) Representative examples of stacked overlays of CXCR3, CXCR5, and CCR6 marker expression gated in UCP-specific polyF triple+ Th1 cells. (I) Overall survival (OS) in patients according to polyF Th1 subsets; *p* value, two-tailed log rank test. (J) Table showing median OS with 95% confidence interval and survival rate at 12 months in overall population and according to polyF Th1 subsets. See also Figures S2 and S3 and Table S3.

To investigate the CD4^+^ Th1 polarization induced by UCPVax, we performed a longitudinal *ex vivo* assessment of IFNγ, TNF-α, and IL-2 production by intracellular cytokine staining (ICS) assay. We confirmed that CD4^+^ T cells, but not CD8^+^ T cells, specifically recognized vaccine peptides (Figures S2A and S2B). We identified three functional Th1 subsets according to the number of Th1-related cytokines produced in response to the peptide vaccine: one, two, or three cytokines referred to as single (singleF Th1), double (polyF double+ Th1), and triple (polyF triple+ Th1) polyF-positive Th1 cells (Figure 2A). The vaccine-induced singleF CD4^+^ Th1 cells mainly produced TNF-α, the polyF double+ Th1 cells were predominantly TNF-α plus IL-2-secreting cells, and the cytokine polyF triple+ Th1 cells concurrently produced IFNγ, TNF-α, and IL-2 (Figure 2B). The cytokine polyF triple+ Th1 cells were expanded after vaccination in 53% of patients compared to 21% and 26% for polyF double+ Th1 and for singleF Th1 cells, respectively (Figure 2C). In some patients, the level of vaccine-induced polyF triple+ Th1 cells exceeded 0.2% of total circulating CD4^+^ T cells (100 cells/mm^3^) and persisted throughout the vaccination period (Figures 2D–2G and S2C). Furthermore, these cells expressed CXCR3, a chemokine receptor associated with Th1 polarization, but lacked CCR6 and CXCR5, which are linked to Th2 and T follicular helper (Tfh) subsets, respectively (Figure 2H).

Additionally, no UCP-specific production of cytokines associated with Th2 (IL-4, IL-5, and IL-13), Th17 (IL-17A), Th9 (IL-9), or Treg (IL-10) polarization was detected after vaccination in either responders or non-responders. These findings confirm that UCPVax selectively induces CD4^+^ T cell polarization toward a Th1 phenotype (Table S3; Figure S3). Notably, patients with polyF triple+ Th1 cells exhibited better clinical outcomes than those with other vaccine-induced Th1 immunotypes, with a median overall survival (OS) of 15 months compared to 8.3 months in patients with single-functional or double-functional Th1 cells (*p* = 0.02) (Figures 2I and 2J).

### Vaccine-expanded CD4^+^ Th1 cells display effector memory and cytolytic features and oligoclonality

**Figure 3:**
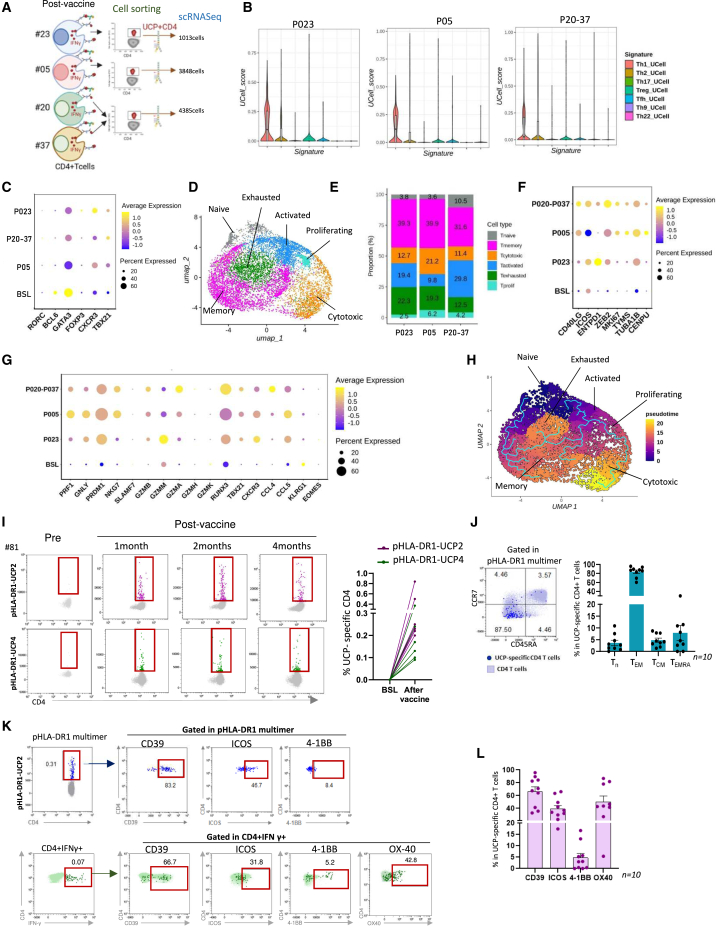
Vaccine-induced CD4^+^ Th1 cells display activated and memory differentiation (A) Experimental design of scRNA-seq of UCP-specific CD4 and bulk CD4 cells performed in four patients, P23, P05, and pooled samples from P20 and P37. (B) UCell scoring of T helper polarization gene signatures in single-cell dataset from bulk CD4 T cells at baseline (BSL; P23) and from vaccine-specific CD4 cells from P23, P05, and P20–P37. (C) Average gene expression of CD4 helper-related transcription factors. (D) Uniform manifold approximation and projection (UMAP) analysis of UCP-specific CD4 T cell differentiation clusters (colors correspond to the cell cluster identified), pooled post-vaccine specific samples from all four patients. (E) Proportion of each cell cluster from post-vaccine samples. (F) Average gene expression of activation and cycling markers. (G) Average gene expression of cytotoxic markers. (H) UMAP visualization by the cell trajectory analysis by monocle3 pseudotime. The scale from 0 to 20 means from the least differentiated state to the most differentiated state. (I) Left, flow cytometry dot plots of pHLA-DR1-UCP2 and pHLA-DR1-UCP4 multimer staining in a representative patient. Right, percentage of pHLA-DR1-UCP2/UCP4 multimer+ CD4^+^ T cells (*n* = 6) at BSL and after vaccine. (J) Representative flow cytometry dot plots showing expression of T cell differentiation markers CCR7 and CD45RA in pHLA-DR1-UCP2 multimer+ cells. Right, histogram (mean ± SD) showing the percentage of naive T (CCR7+CD45RA−), T effector memory (CCR7−CD45RA−), T central memory (CCR7+CD45RA+), and terminally differentiated effector memory T cells TEMRA (CCR7−CD45RA+) cells in pHLA-DR1-UCP2 multimer+ cells or ICS (*n* = 10 patients). (K) Top, representative dot plots of activation markers CD39, ICOS, 4-1BB, and OX40 expression gated on pHLA-DR1 multimer+ cells and bottom on IFNγ+ UCP-specific CD4^+^ T cells by *ex vivo* ICS assay. (L) Percentage of CD39, ICOS, 4-1BB, and OX40 + UCP-specific CD4^+^ T cells by ICS or pHLA-DR1 multimer (*n* = 10). Mean ± SD See also Figure S5.

**Figure 4:**
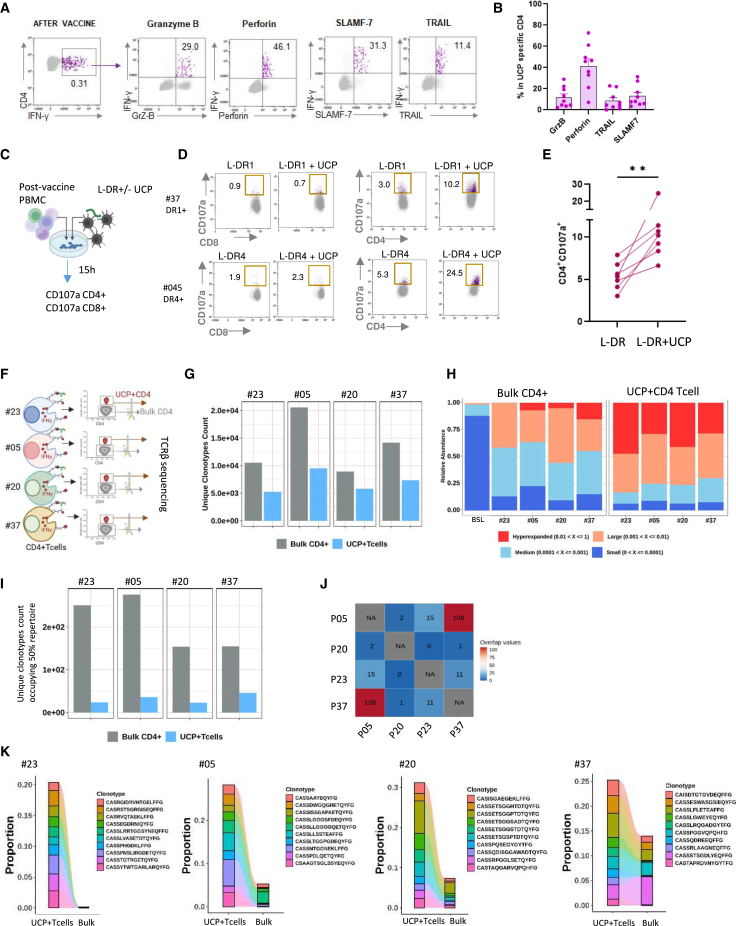
UCP-specific CD4^+^ T cells are cytolytic and oligoclonal (A) Flow cytometry dot plots showing expression of cytotoxic makers granzyme B, perforin, SLAMF-7, and TRAIL in IFNγ+ UCP-specific CD4^+^ T cells in representative patients. (B) Percentage of each cytotoxic marker+ in vaccine-expanded specific CD4^+^ T cells, mean ± SD (*n* = 9 patients). (C) CD107a degranulation assay. Post-vaccine PBMCs were co-cultured with HLA-DR-matched L-DR-cell line loaded or not with UCP2/4 during 15 h before CD107a staining. (D) Example flow cytometry dot plot of CD107a expression in two representative patients. (E) Percentage of CD107a+ CD4^+^ T cells after co-culture (*n* = 9 patients). Mann-Whitney test. (F) Experimental design of TCR-β sequencing of UCP-specific CD4 and bulk CD4 cells performed in four patients: P23, P05, P20, and P37. (G) Number of total clonotypes in UCP-specific CD4 and bulk CD4 fractions. (H) Relative abundance of small, medium, large, and hyperexpanded clonotypes in UCP-specific CD4 and bulk CD4 fractions at baseline. (I) Number of clonotypes occupying 50% of the repertoire. (J) Repertoire overlaps between the patients’s UCP-specific T cells. (K) Tracking of most frequent (top 10) clonotypes in UCP-specific CD4 and bulk CD4 fractions. See also Figure S5.

To deeply characterize vaccine-induced CD4^+^ Th1 cells, we performed single-cell RNA sequencing (scRNA-seq) of UCP-specific CD4^+^ T cells sorted from peripheral blood mononuclear cells (PBMCs) in four vaccinated patients using an IFNγ secretion-detection assay (Figure 3A). Bulk CD4 from PBMCs collected before vaccination in one patient was used as a control. Compared to the baseline, violin plots confirmed the predominance of the Th1 signature of vaccine-expanded UCP-specific CD4^+^ T cells (Figures 3B and S4A). Accordingly, vaccine-specific CD4^+^ T cells exhibited high expression of Th1 markers, such as CXCR3 and TBX21, while showing low or no expression of transcription factors associated with Th2 (*GATA3*), Treg (*FOXP3*), or Th17 (*RORC*) differentiation (Figure 3C). Unsupervised clustering of UCP-specific CD4^+^ T cells showed six different transcriptional clusters enriched in effector subsets, including activated, proliferating, cytotoxic, and memory, compared to naive and exhausted clusters (Figures 3D, 3E, and S4B). Transcripts associated with activation (*CD40L*, *ICOS*, and *ENTPD*), proliferation (*MKI67*, *ZEB2*, and *TUBA1B*), and cytotoxicity (*PRF1*, *GNLY*, *GZMB*, *GZMM*, *NKG7*, *PRDM1*, and *RUNX3*) were upregulated in vaccine-induced CD4^+^ T cells compared to baseline (Figures 3F, 3G, and S4C). Pseudotime analysis of UCP-specific cells revealed two main trajectories: one from CD4^+^ T naive-like going through memory and exhausted clusters and the second from activated CD4^+^ T cells going through proliferating and cytotoxic, with one branch progressing through memory and exhausted clusters (Figure 3H). Next, data were confirmed using *ex vivo* HLA-DR1 multimer staining, which revealed that vaccine-expanded CD4^+^ T cells were predominantly effector memory cells expressing activation markers such as ICOS, 4-1BB, and OX40. Additionally, these cells displayed CD39 expression, indicating their antigen-experienced status (Figures 3I–3L). The cytolytic signature was further confirmed at the protein level, showing the expression of cytotoxic factors such as granzyme B, perforin, SLAMF-7, and TRAIL (Figures 4A, 4B, and S5A). Furthermore, we measured cytolytic activity using a CD107a degranulation assay after the co-culture of post-vaccine PBMCs with HLA-DR matched-target cells loaded with UCP (Figure 4C). Results showed that vaccine-induced CD4^+^ T cells, but not CD8^+^ T cells, degranulated in the presence of corresponding UCP-loaded HLA-DR+ target cells (Figures 4D and 4E). Overall, activation, memory, and cytotoxic markers were especially expressed on vaccine-expanded CD4^+^ T cells in patients’ peripheral blood (Figure S5B).

To study the individual clonotypes in vaccine-specific CD4^+^ T cells, we performed T cell receptor (TCR)-β sequencing analysis from PBMCs in the same patients as for scRNA-seq (Figure 4F). A TCR repertoire bias with clonal restriction was observed in UCP-specific CD4^+^ T cells compared to bulk CD4 fractions (Figure 4G). Increased clonality was found in UCP-specific CD4^+^ T cells compared to bulk CD4^+^ T cells, characterized by a higher proportion of hyperexpanded clonotypes compared to small and medium clonotypes, suggesting a selective expansion of specific T cell clones following vaccination (Figure 4H). The presence of large clonotypes in the bulk CD4^+^ fraction, compared to the baseline, may be attributed to our cell sorting method. Although most vaccine-specific CD4^+^ T cells produced IFNγ, some also secreted other Th1 cytokines, such as IL-2 and TNF-α, which could contribute to this background signal in the bulk CD4^+^ fraction. In addition, a small number of clonotypes occupied 50% of the repertoire, indicating an oligoclonal expansion (Figure 4I), and we observed a low TCR repertoire overlap among patients, except for patients #05 and #37 (Figure 4J). Clonotype tracking further confirmed that the most frequent vaccine-induced CD4^+^ clonotypes (top ten) were rarely detected in the bulk CD4^+^ T cells (Figure 4K).

### Targeting CD4^+^ T cells with UCPVax promotes Ab response and epitope spreading

**Figure 5:**
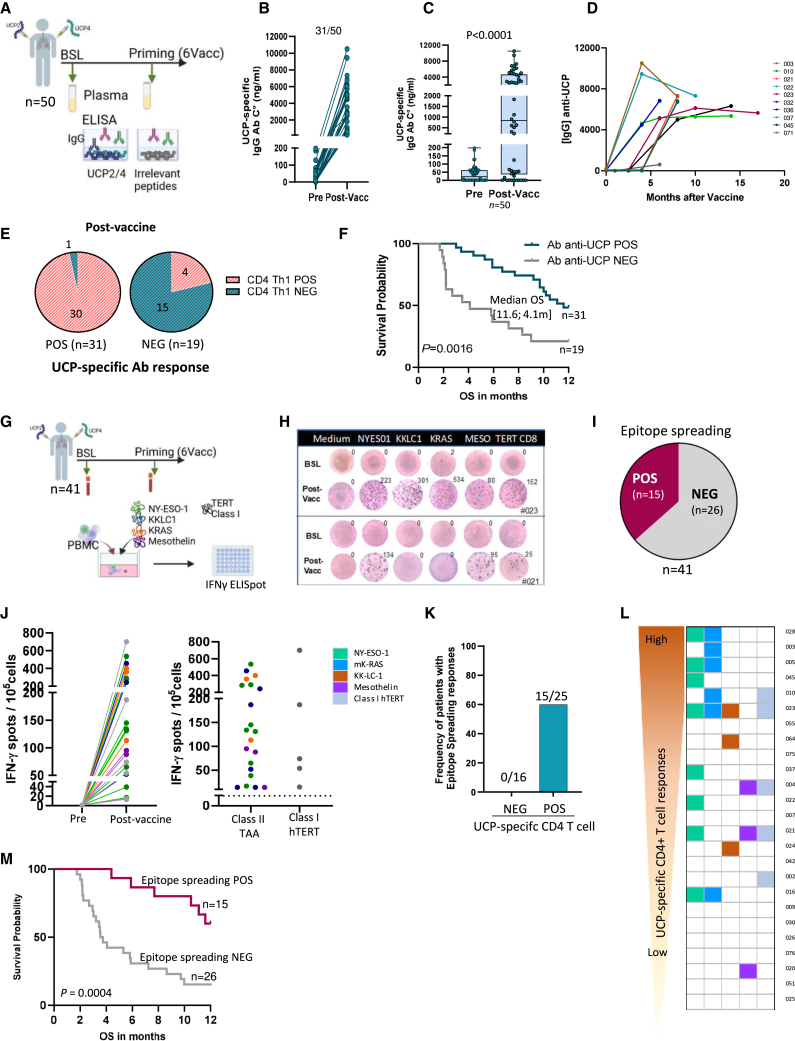
Targeted CD4^+^ T cells with UCPVax promotes specific antibody response and epitope spreading (A) Schematic design of vaccine-induced antibody (Ab) response by ELISA in plasma. (B) UCP-specific IgG1 Ab titer (ng/mL) before (pre) and after priming vaccinations (post-vacc) (*n* = 50). (C) Boxplot showing anti-UCP IgG1 titer Ab (ng/mL) pre- and post-vaccinations (*n* = 50). Data are presented as median ± 1^st^ and 3^rd^ quartiles. Mann-Whitney test. (D) Graphs showing evolution of anti-UCP IgG response during vaccination in 10 representative patients. (E) Pie charts representing the distribution of patients with positive Ab response (POS; *n* = 31) on the left and without Ab response (NEG; *n* = 19) on the right according to the UCP CD4^+^ Th1 response by ELISpot; positive in red and negative in blue. (F) Overall survival (OS) according to anti-UCP Ab response (*n* = 50). *p* value, two-tailed log rank test. (G) Schematic design of epitope-spreading assessment pre- and post-vaccination by IFNγ ELISpot after 6 days. IVS of PBMCs with mixture of MHC class I and II peptides derived from indicated tumor-associated antigens (TAAs) (*n* = 41). (H) Representative IFNγ spot wells from two patients positive for epitope spreading. (I) Pie chart representing number of patients with (POS) or without (NEG) epitope-spreading induction. (J) Left, expansion of T cells against indicated TAA in the IFNγ ELISPOT assay. Right, distribution of specific T cells against class II epitope from TAA and class I peptides from hTERT. (K) Frequency of patients with epitope spreading according to anti-UCP CD4^+^ T cell response after vaccination. (L) Heatmap showing diversity of epitope-spreading response according to intensity of anti-UCP CD4^+^ T cells (*n* = 25). (M) OS according to epitope spread response. *p* values, two-tailed log rank test.

Basically, activated CD4^+^ T cells provide help for B cell differentiation and class switching.^43^ Thus, we investigated the ability of this helper vaccine to stimulate specific Ab production. To this end, immunoglobulin (Ig)G Ab response was measured in the plasma of vaccinated patients using a customized ELISA (Figure 5A). The results showed that 59% (31/50) of patients developed a UCP-specific IgG Ab response after priming vaccinations. Most of them (61%, 19/31) displayed a high titer of anti-UCP Ab (>1,000 anti-UCP IgG ng/mL), which was still detectable over time during the boost vaccination (Figures 5B–5D). Among vaccinated patients, all except one (30/31) who developed anti-UCP IgG Abs also exhibited a concurrent UCP-specific CD4^+^ Th1 response, compared to only 4 patients in the group without a detectable Ab response (4/19) (Figure 5E). In contrast, no UCP-specific Abs were detected in healthy controls (*n* = 25) or non-vaccinated patients with cancer (*n* = 40) (data not shown). These findings suggest that vaccine-induced CD4^+^ T cells play a key role in promoting UCP-specific Ab production by B cells. The induction of the UCP-specific Ab response was associated with significant improvement of OS: the median OS was 11.6 versus 4.1 months in the absence of an Ab response (Figure 5F).

Next, we investigated the ability of UCPVax to elicit the epitope spread response. Epitope spreading is an immunological process associated with cancer vaccine efficacy and characterized by the diversification of T cell response against epitopes that are different from the originally targeted antigen.^44^ Epitope spread responses were measured by IFNγ ELISpot after short *in vitro* stimulation with a mixture of HLA class II-binding epitopes derived from various TAAs expressed in lung cancer, such as NY-ESO-1, KK-LC-1, mesothelin, and mK-RAS, and with HLA class I peptides from hTERT (Figure 5G). The detection of a *de novo* T cell response against any tested tumor antigen after vaccination was defined as an epitope spreading, which was observed in 15 out of 41 evaluated patients (36.5%) (Figures 5H and 5I). The epitope spread response was directed against both HLA class II epitopes derived from non-vaccinated antigens (intermolecular spreading) and HLA class I epitopes derived from hTERT, the vaccinated antigen (intramolecular spreading) (Figure 5J). Notably, this T cell diversification process was exclusively observed in patients with vaccine-induced CD4^+^ Th1 responses (Figure 5K) and was more extensive in patients with a strong CD4^+^ T cell response (Figure 5L). Patients with an epitope-spreading response demonstrated improved OS, with the median OS not reached in the epitope-spreading-positive group compared to 3.6 months in the epitope-spreading-negative group (*p* = 0.0004) (Figure 5M).

### The triad of immune responses triggered by UCPVax promotes optimal tumor control

**Figure 6:**
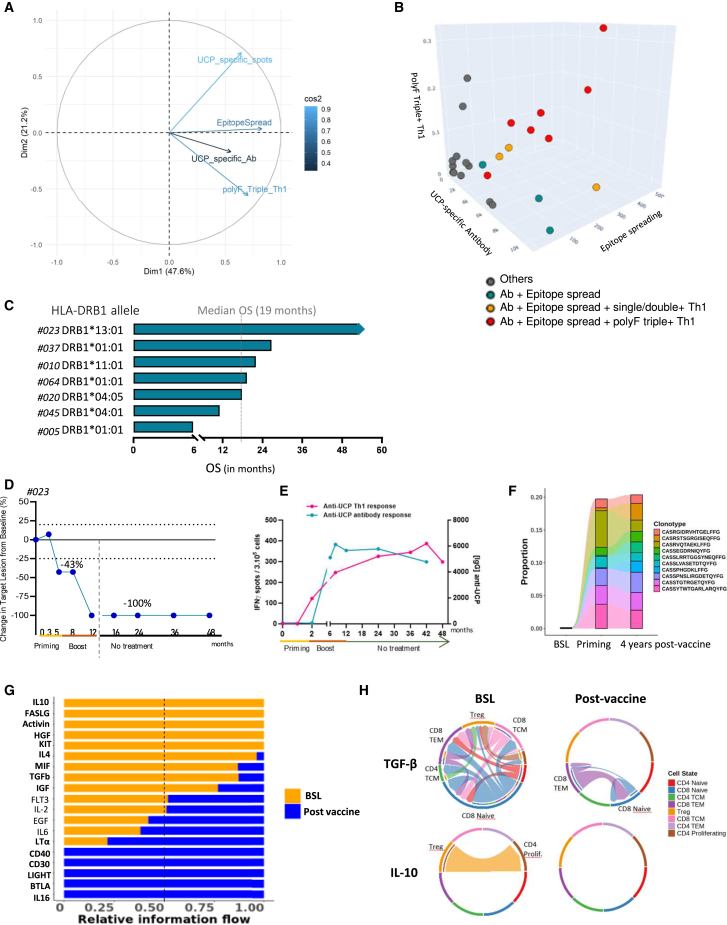
UCPVax-mediated immune triad is associated with optimal tumor control (A) Unsupervised principal-component analysis (PCA), including UCP-specific CD4^+^ T cells (IFNγ spots), polyF triple+ Th1 cells, anti-UCP Ab response, and epitope spreading. (B) 3D clustering graph (*k*-means) showing individual patient according to vaccine-induced immune response subtype. (C) Graph showing HLA-DR alleles and OS of the 7 patients with vaccine-induced immune triad. (D) Change in target lesion from baseline (BSL) evaluated by RECIST1.1 in one patient with complete response (P23). (E) Kinetic of vaccine-induced UCP-specific CD4 and antibody responses since inclusion in P23. (F) Tracking of the most frequent clonotype (top 10) of UCP-specific clones from BSL, priming, and 4.5 years post-vaccine in P23. Red: preserved clonotypes along the time. (G) Predominant signaling pathway on T cells pre- versus post-vaccination by CellChat. Top signaling pathways enriched at BSL (orange) and more enriched after vaccination (blue). In bold: pathways that are statistically significant. (H) Chord diagram showing the cell-cell communication mediated by TGF-β and IL-10 signaling. Arrows and edge color indicate direction (source: target). Edge thickness indicates the sum of weight key signals between populations. See also Figure S6 and Tables S4 and S5.

To evaluate the contribution of each type of UCPVax-mediated immune response in tumor control, we performed a principal-component analysis (PCA). Figure 6A shows that the three immune factors considered in the PCA, cytokine polyF triple+ Th1 cells, UCP-specific Ab response, and epitope spreading, were grouped together, indicating their positive correlation. However, the induction of polyF triple+ Th1 cells and epitope-spreading response had a major contribution compared to the Ab response, including in multivariate analysis (Table S4). Next, we assessed whether baseline immunological parameters that influence overall vaccine immunogenicity could also affect the vaccine-induced immune triad. As expected, we found that high circulating levels of IL-6 and IL-8 at baseline were associated with the absence of this immune triad, suggesting that a high inflammatory status limits the immunogenicity of UCPVax (Table S5).

Both PCA and three-dimensional correlation analysis identified a group of patients (*n* = 7) who presented the vaccine-induced immune triad in multiple HLA-DR restriction contexts, and their median OS reached 19 months (Figures 6B, 6C, and S6). Among this group, we observed two objective responses, including one complete response (patient #23). This patient had KRAS-mutated metastatic adenocarcinoma NSCLC, enrolled in June 2019 after three previous lines of treatment, and remained relapse free without any subsequent treatment 5 years after the last vaccination (Figure 6D). This long-term remission was associated with sustained and strong UCPVax-induced cellular and humoral responses detectable *ex vivo* over time (Figures 6E and 6F). Furthermore, longitudinal single-cell transcriptomic analysis of total blood T cells from this patient showed that molecular interactions at baseline were enriched in pathways related to tumor immune escape, such as IL-10 and transforming growth factor β (TGF-β), which shifted after vaccination toward pathways involved in T cell homeostasis, and function, such as lymphotoxin α (LTα), CD40, and IL-16, after UCPVax vaccination (Figures 6G and 6H), suggesting that the vaccine remodeled the molecular interactions of patients’ CD4^+^ T cells, which could explain the robust and durable hTERT-specific CD4^+^ Th1 response detectable over time in this patient. Collectively, our results indicate that the UCPVax-mediated immune triad was correlated with optimal tumor control in patients with advanced NSCLC.

## Discussion

The induction of antitumor CD4^+^ T cells has gained considerable interest for cancer vaccine efficacy during the past decade.^8^^,^^45^ However, few cancer vaccines have been designed to exclusively target CD4^+^ T cells so far.

Our study demonstrated that CD4^+^ T cells targeting vaccine UCPVax stimulate multifaceted and sustained cellular and humoral antitumor immunity in patients with advanced NSCLC. This vaccine expanded robust and sustained UCP-reactive CD4^+^ T cells displaying Th1 polarization, in agreement with our previous findings in mice.^36^^,^^37^ In contrast, we demonstrated that multiple injections of UCPVax do not commit CD4^+^ T cells toward Th2, Th17, or Treg polarization, which rules out any risk of inducing a pro-tumoral immune response. Given the plasticity of CD4^+^ T cells,^26^^,^^27^ directly targeting these cells with a helper peptide vaccine carries the risk of promoting undesired immune responses, as recently described.^28^ This challenge may explain the limited clinical translation of CD4-targeted vaccine strategies. To minimize this risk, it is crucial to carefully select tumor-reactive helper peptides that can induce a Th1-polarized immune response,^46^ and UCPVax has this characteristic.

This vaccine stimulates various subsets of cytokine polyF CD4^+^ Th1 cells, especially the polyF triple+ Th1 cells that simultaneously produce IFNγ, TNF-α, and IL-2, and their induction is associated with improved clinical outcomes. Cytokine polyfunctionality is a well-established concept in T cells, and their capacity to produce multiple Th1 cytokines concurrently has been associated with enhanced immunological control, particularly in infectious diseases.^47^ Recent studies also reported the involvement of cytokine polyF T cells in anticancer vaccine efficacy, including neoantigen vaccines, underlining the interest in stimulating these high-quality T cells.^4^^,^^6^^,^^48^^,^^49^

Beyond their cytokine polyF capacity, we found that UCPVax-expanded CD4^+^ T cells express cytotoxic molecules as well as transcription factors involved in cytotoxic CD4^+^ T cell differentiation.^22^ They also degranulated against target cells *in vitro* after MHC class II/peptide recognition, supporting their cytolytic capacity. These data support the fact that the induction of cytotoxic CD4^+^ T cells contributes to the efficacy of the cancer vaccine, as recently reported.^49^^,^^50^^,^^51^ Although we were not able to investigate direct recognition of autologous cancer cells, the ability of UCPVax to trigger cytolytic CD4^+^ T cells suggests that they can contribute to cancer cell killing in the TME, as previously reported.^52^ It has been shown that cytotoxic CD4^+^ T cells can directly kill MHC class II+ target cells through various mechanisms, including the release of perforin and granzyme.^22^^,^^53^ Because most tumors are MHC class II negative, we assume that IFNγ produced by vaccine-specific CD4^+^ T cells can upregulate HLA-DR expression in tumor cells, making them susceptible to lysis. Conversely, cytotoxic CD4^+^ T cells can also kill tumor cells in an MHC class II-independent fashion through a mechanism involving dendritic cells (DCs) and innate cells in the TME.^54^

One fundamental role of CD4^+^ T cells is to provide help to B cells for Ab production,^43^ but little is known about the role of Abs induced by cancer vaccines, particularly with peptide vaccines. We report here the ability of the UCPVax to stimulate the IgG Ab response directed against hTERT. This UCP-specific IgG response was strongly correlated to the CD4^+^ T cell response induced by the vaccine, suggesting their synergistic interaction. Our result is in line with previous studies showing that long peptide-based vaccination promoted the Ab response associated with improved clinical outcomes.^55^^,^^56^ However, the mechanism of action of anti-peptide Ab is not well established. UCP-specific Abs are not expected to recognize tumor cells because these peptides are derived from an intracellular protein. Nevertheless, these Abs could have implications for vaccine immunogenicity; for example, Abs could opsonize the peptide vaccine in immune complexes, which could reduce peptide degradation and increase uptake and presentation by DCs to enhance antitumor T cell responses.^57^^,^^58^ Thus, future therapeutic cancer vaccines will likely benefit from stimulating B cells as well as T cells, which is what CD4-targeted vaccines provide.

Another aspect of this vaccine relies on its ability to elicit robust antigen spreading. This immune phenomenon consists of an increase or *de novo* induction in circulating T cells specific to tumor antigens that were not included in the vaccine and contributes to T cell diversification.^44^^,^^59^ Here, we detected, in blood, T cell responses against a broad range of MHC class I and class II tumor epitopes, suggesting the ability of UCPVax to enhance both CD4^+^ and CD8^+^ T cell diversification in vaccinated patients. This immune process was exclusively induced in immune responders to UCPVax and correlated with the quality of vaccine-specific CD4^+^ T cells. Mechanistically, the induction of antigen spreading relies on uptake and cross-presentation by DCs of antigens released from tumor cell lysis by vaccine-induced T cells.^44^ Thus, we suppose that UCPVax-expanded cytotoxic CD4^+^ T cells, together with the vaccine-induced Ab response, may be involved in this process. Epitope spreading was considered a surrogate indicator of tumor lysis induced by the vaccine-expanded immune cells and was associated with cancer vaccine efficacy.^2^^,^^44^^,^^60^ This clinical benefit associated with epitope-spreading induction was also observed in our study, underlining the interest of a CD4^+^ T cell-stimulating vaccine to promote epitope spreading.^25^

Overall, the stimulation of CD4^+^ T cells by UCPVax vaccination resulted in three main types of immune responses, including cytokine polyF Th1 cells, helper humoral immunity, and epitope spreading in patients with NSCLC. The induction of this UCPVax-related immune triad was associated with the best clinical outcome observed in this study since the median OS exceeded 15 months in these patients, compared to 9 months in the overall cohort. Although the number of patients who developed this immune triad is limited, these results are very encouraging in the context of refractory metastatic disease without any co-therapy. In addition, the vaccine-mediated immune triad persisted *in vivo* and was still detectable 4 years after vaccination in one patient who achieved complete remission, in favor of a protective memory immunity. In line with our finding, a recent report showed a long-term clinical benefit in patients with melanoma who were previously vaccinated 10 years ago with a melanoma CD4^+^ helper peptide vaccine as adjuvant therapy,^34^ highlighting the importance of developing CD4^+^ T immunological memory to improve cancer vaccine efficacy.^61^

Telomerase (hTERT), the shared tumor antigen targeted by this vaccine, is a hallmark of cancer cells.^38^ This enzyme maintains telomere length in dividing cells and is the predominant mechanism in malignant cells to escape telomere-dependent cell death. Consequently, hTERT expression has been observed in all studied cancers (>85% of all cancers).^39^ We previously reported a naturally occurring CD4^+^ T cell response against hTERT in several cancers, including NSCLC, and this presence was associated with a good prognosis.^62^^,^^63^ Although hTERT is not a neoantigen, its critical role during oncogenesis considerably limits the immune escape by antigenic loss phenomenon. Therefore, hTERT represents a prototype of a universal and attractive target for cancer vaccines.^64^ Therefore, UCPVax is currently being evaluated in various cancers, such as glioblastoma, cervical cancer, and hepatocellular carcinoma, in combination with chemotherapy or checkpoint inhibitors.^65^^,^^66^

The search for baseline factors that could predict the response to the vaccine revealed that blood inflammatory status plays a role, with high levels of IL-6 or IL-8 observed in non-responders. Additionally, we found an association between the duration of prior anti-PD-1 therapy and immune response to the vaccine. However, these findings require further validation in ongoing trials before they can be used as predictive biomarkers.

In conclusion, UCPVax vaccination targets CD4^+^ T cells, promoting highly functional CD4^+^ Th1 cells that work synergistically with the Ab response and antigen spreading to enhance clinical outcomes. This vaccine can be used as a universal peptide platform to provide help for cancer vaccines, including personalized approaches, regardless of HLA genotype or cancer type.

### Limitations of the study

The main limitation of this study is the lack of tumor tissue analysis to assess immunological changes in the TME induced by vaccination. Such evaluation would require invasive biopsies, which are particularly challenging in this population of heavily pretreated metastatic patients. For single-cell and TCR sequencing, sorting of vaccine-induced CD4^+^ T cells using peptide-MHC class II multimers was not feasible, although it would have been more relevant than IFNγ-based cell sorting. The potential baseline biomarkers identified must be taken with caution, as the majority of patients were enrolled after receiving third-line treatment.

## Resource availability

### Lead contact

Further information and requests for resources and reagents should be directed to and will be fulfilled by the lead contact, Prof. Olivier Adotévi (olivier.adotevi@univ-fcomte.fr).

### Materials availability

This study did not generate new unique reagents.

### Data and code availability

• scRNA-seq and TCR-seq data have been deposited at the Gene Expression Omnibus (GEO) and are publicly available under GEO: GSE271543 and GEO: GSE271545 for scRNA-seq and TCR-seq, respectively.
• This paper does not report original code.
• Any additional information required to reanalyze the data reported in this paper is available form the lead contact upon request.

## Acknowledgments

The authors thank all patients and their families who contributed to this study. We thank the medical doctors, nurses, and research assistants from the Oncology Department and Pneumology Department of University Hospital of Besançon and nurses from the clinical investigational center CIC-1431 (Sinetic) of the University Hospital of Besançon; Georges François Leclerc Cancer Center of Dijon; the Oncology Department and CIC 1427 of AP-HP Saint Louis Hospital of Paris; the Pneumology Department of Strasbourg University; and the Pneumology Department of E. Muller Hospital of Mulhouse for their contributions. Many thanks to the HLA laboratory of the French blood bank (EFS Besançon) for HLA genotyping. We thank the Pharmacy Department of University Hospital of Besançon for the production, qualification, and distribution of the vaccine and the delegation to clinical research and innovation (DRCI) of University Hospital of Besançon for their support. This work was supported by grants from the National Institute of Cancer (PHRC-K 13-063) and the cancer research foundations 10.13039/501100004097Fondation ARC and 10.13039/501100004099Ligue Contre le Cancer.

## Author contributions

Conceptualization, O.A.; methodology, O.A., C.L., R.G., and C.J.; validation, O.A., C.L., L.B., R.G., and B.N.; investigation, L.B., M.M., L.Q., P.G., E.G., A.R., and R.L.; resources, P.G., F.G., and C.J.; formal analysis, B.N., E.S., C.L., Y.G., A.M., and D.V.; data curation, A.M. and D.V.; writing—original draft, C.L., B.N., L.B., and O.A.; writing—review & editing, C.L., E.S., R.L., F.G., R.G., Y.G., C.J., C.B., and O.A.; supervision, O.A.; project administration, M.J.; funding acquisition, O.A.

## Declaration of interests

O.A. is an inventor on a patent related to this work.

## Declaration of generative AI and AI-assisted technologies in the writing process

During the preparation of this work, the authors used ChatGPT for English proofreading. After using this tool/service, the authors reviewed and edited the content as needed and take full responsibility for the content of the publication.

## STAR★Methods

### Key resources table

**Table:** 

REAGENT or RESOURCE	SOURCE	IDENTIFIER
**Antibodies**		

Alexa Fluor 488 anti-human CD4	Biolegend	Cat#300519; RRID:AB_389311
PE anti-human IL-2	Biolegend	Cat#500307; RRID:AB_315094
PE-Cyanine7 anti-human CD8	Biolegend	Cat#344712; RRID:AB_2044008
APC anti-human IFN-γ	Biolegend	Cat#502512; RRID:AB_315237
TNFα-AF700	Beckman Coulter	Cat#B76295; RRID:AB_2561315
Pacific Blue Mouse Anti-Human CD3	BD Biosciences	Cat#558117; RRID:AB_397038
eFluor506 Fixable Viability Dye	eBioscience	Cat#65-0866-14
PE/Dazzle 594 anti-human CD39	Biolegend	Cat#328224; RRID:AB_2564319
APC anti-human CD319 (CRACC/SLAMF7)	Biolegend	Cat#331810; RRID:AB_2564333
Alexa Fluor 700 anti-human CD3	Biolegend	Cat#300424; RRID:AB_493741
APC/Cyanine7 anti-human CD45RA	Biolegend	Cat#304128; RRID:AB_1070888
Brilliant Violet 421 anti-human/mouse/rat CD278 (ICOS)	Biolegend	Cat#313523; RRID:AB_2562538
Brilliant Violet 650 anti-human CD197 (CCR7)	Biolegend	Cat#353234; RRID:AB_2563867
BV786 Mouse Anti-Human CD137 (4-1BB)	BD Biosciences	Cat#741000; RRID:AB_2740623
PE anti-human IFN-γ	Biolegend	Cat#502509; RRID:AB_315234
PerCP-Cy5.5 Mouse Anti-Human CD8	BD Biosciences	Cat#565310; RRID:AB_2687497
Brilliant Violet 605 anti-human/mouse/rat CD278 (ICOS)	Biolegend	Cat#313537; RRID:AB_2687078
Alexa Fluor 488 Mouse Anti-Human Perforin	BD Biosciences	Cat#563764; RRID:AB_2738411
PE anti-human CD319 (CRACC/SLAMF7)	Biolegend	Cat#331806; RRID:AB_2239190
PE-CF594 Mouse Anti-Human Granzyme B	BD Biosciences	Cat#562462; RRID:AB_2737618
PE-Cy7 Mouse Anti-Human CD3	BD Biosciences	Cat#563423; RRID:AB_2738196
eFluor780 Fixable Viability Dye	eBioscience	Cat#65-0865-14
BV510 Mouse Anti-Human CD4	BD Biosciences	Cat#562970; RRID:AB_2744424
Brilliant Violet 605 anti-human CD134 (OX40)	Biolegend	Cat#350028; RRID:AB_2629633
BV650 Mouse Anti-Human CD253 (TRAIL)	BD Biosciences	Cat#743721; RRID:AB_2741697
Brilliant Violet 785 anti-human CD8	Biolegend	Cat#344740; RRID:AB_2566202
7-AAD Viability Dye	Beckman Coulter	Cat#IM3422
PE Mouse Anti-Human CD107a	BD Biosciences	Cat#555801; RRID:AB_396135
Alexa Fluor 488 anti-human CD183 (CXCR3)	Biolegend	Cat#353710; RRID:AB_10962442
PE Anti-Human IL-4	BD Biosciences	Cat#554485; RRID:AB_395424
Anti-human IL-17A-PB	Beckman Coulter	Cat#B76266; RRID:AB_961392
Brilliant Violet 605 anti-human CD185 (CXCR5)	Biolegend	Cat#356930; RRID:AB_2566227
Brilliant Violet 650 anti-human CD196 (CCR6)	Biolegend	Cat#353426; RRID:AB_2563869
Brilliant Violet 785 anti-human CD3	Biolegend	Cat#344740; RRID:AB_2566202
eFluor506 Fixable Viability Dye	eBioscience	Cat#65-0866-14
APC Mouse Anti-Human CD3	BD Biosciences	Cat#555335; RRID:AB_398591
BV421 Mouse Anti-Human CD4	BD Biosciences	Cat#562424; RRID:AB_11154417
CD127 Monoclonal Antibody PerCP-Cyanine5.5	eBioscience	Cat#45-1278-42; RRID:AB_10670349)
PE-Cy7 Mouse Anti-Human CD25	BD Biosciences	Cat#557741; RRID:AB_396847
Alexa Fluor 647 anti-human FOXP3	Biolegend	Cat#320214; RRID:AB_492984
Pacific Blue anti-human CD197 (CCR7)	Biolegend	Cat#353210; RRID:AB_10918984
Brilliant Violet 605™ anti-human CD45RA	Biolegend	Cat#304134; RRID:AB_2563814
BV786 Rat Anti-Human IL-10	BD Biosciences	Cat#564049; RRID:AB_2738563

**Biological samples**		

PBMC, serum, plasma from NSCLC vaccinated patients	CHU Besançon	UCPVax trial, NCT02818426

**Chemicals, peptides, and recombinant proteins**		

PepMix Human NY-ESO-1	JPT	Cat#PM-NYE
mK-RAS	This manuscript	N/A
Mesothelin	This manuscript	N/A
15-mers sequences KKLC1	ProImmune	N/A
PepMix HIV	JPT	Cat#PM-HIV-ENV
ProMix™ hTERT Peptide Pool	ProImmune Ltd	Cat#PX-TERT
MHC Class II multimers PE pMHC-UCP2/HLADR1, BV605 pMHC-UCP4/HLADR1	Peptide and Tetramer Core Facility, UNIL-CHUV, Epalinges, Switzerland)	N/A

**Critical commercial assays**		

Cytometric Bead Array (CBA) Flex Set	BD Biosciences	N/A
Fixation/Permeabilization Solution Kit with BD GolgiPlug	BD Biosciences	Cat#555028
Protein Transport Inhibitor (Containing Monensin)	BD Biosciences	Cat#554724
Stain Buffer (FBS)	BD Biosciences	Cat#554656
Human FoxP3 Buffer Set	BD Biosciences	Cat#560098
Chromium Next GEM Single cells 3′ Kit v3.1	10X Genomics	Cat#PN-1000269
Chromium Next GEM Single cells 5′ Kit v2.4	10X Genomics	Cat#PN-1000265
Dual index kit TT set A	10X Genomics	Cat#PN-1000215
SPRI select Reagent kit	Beckman coulter	Cat#B23317
CD4^+^ T Cell Isolation Kit	Miltenyi	Cat#130-096-553
IFN-γ Secretion Assay – Detection Kit (PE), human	Miltenyi	Cat#130-054-202
Dynabeads mRNA Purifications Kits	ThermoFisher	Cat#A33562
Human IFN gamma ELISpot Set	Diaclone	Cat#856051020P
ELISpot Kit Pro Human IFN-γ (HRP)	Mabtech	Car#3420-2HST-10

**Deposited data**		

scRNA sequencing data	This paper	GEO: GSE271543
TCR sequencing data	This paper	GEO: GSE271545

**Software and algorithms**		

R software version 4.2.3	R Core Team (2021)	http://www.R-project.org/
cellranger	https://doi.org/10.1038/ncomms14049	https://www.10xgenomics.com/support/software/cell-ranger
Seurat v.4.9.9 package	Satija et al., 2015	https://satijalab.org/seurat/
Azimuth v.0.4 package	Hao et al., 2021	https://satijalab.github.io/azimuth/articles/run_azimuth_tutorial.html
cellchat v. 1.5.0	https://doi.org/10.1038/s41467-021-21246-9	https://github.com/sqjin/CellChat
UCell v 2.6.2	https://doi.org/10.1016/j.csbj.2021.06.043	https://bioconductor.org/
monocle3 v.1.3.7	Trapnell C.et al.	https://bioconductor.org/
escape v.1.12.0	https://doi.org/10.1038/s42003-020-01625-6	https://bioconductor.org/
MiXCR	https://doi.org/10.1038/nmeth.3364	https://mixcr.com/
immunarch v.0.9		https://immunarch.com/
GraphPad Prism V.10.0.2	GraphPad Software, California, USA	http://www.graphpad.com
Kaluza software version 2.1		N/A
ImmunoSpot software version 5.1	Cellular Technology Ltd, Germany	N/A
FCAP array version 3	BD Biosciences	N/A
NetMHCpan (version 4.0)	DTU Health Tech Marty et al.^34^^,^^62^	http://www.services.healthtech.dtu.dk

### Experimental model and study participant details

#### Patients and trial design

Patients with metastatic NSCLC with relapse after two previous lines including platinum-based chemotherapy and anti-PD-(L)1 therapy were included in this multicenter phase Ib/IIa UCPVax trial (NCT02818426). Trial was conducted in five French cancer centers from April 2016 to March 2022. Patients were aged 62.8 to 72.2 years, comprising 41 men and 19 women, had an Eastern Cooperative Oncology Group performance status of 0 or 1 and did not receive chemo-immunotherapy combination. Additional clinical information is presented in Table S1. Main exclusion criteria included symptomatic brain metastases, active autoimmune diseases, administration of systemic corticoids or other immunosuppressive drugs. Patients were required to have measurable disease. The study was performed in accordance with the Declaration of Helsinki and Good Clinical Practice guidelines. All patients provided written informed consent. Approval of the protocol was obtained from an independent ethics committee (CPP Est-II: 15/10/2015) and French Drugs Safety Agency (01/09/2015).

### Method details

#### Vaccination schedule

UCPVax vaccine is composed of two hTERT-derived 15 amino acid long peptides called UCP2 and UCP4.^41^ Three doses (0.25, 0.5 and 1mg) of each helper peptide was emulsified in the adjuvant Montanide ISA-51 VG (Seppic Inc, Courbevoie, France) before injection subcutaneously in rotative sites as reported.^41^ UCPVax was administrated on a schedule of six priming doses given weekly (days 1, 8, 15, 29, 36, and 43) followed by a booster vaccination every 8 weeks for a maximum of 12 months.

#### Tumor assessment

Clinical status was assessed during patient visits by a thoracic oncologist. CT scan was performed at baseline and every 8-9 weeks and tumor size were determined according to RECIST v1.1. Survival data were censored at the date of last follow-up. Overall survival (OS) was estimated from the date of inclusion to the date of death from any cause. Progression free survival (PFS) was calculated from the date of inclusion to the date of progression or death from any cause, or the date of last follow-up at which point data were censored. The data cutoff date was March 13, 2024.

#### Blood samples

Patient samples (Peripheral Blood mononuclear cells (PBMCs), sera, plasma and ctDNA) were collected at different time point along the study. PBMCs were isolated by density gradient separation on Ficoll Unisep tube (Eurobio, France) and used in *ex vivo* assays. A part of PBMCs were cryopreserved in CryoStor medium (Sigma Aldrich, France) and stored in liquid nitrogen until use. Sera, plasma and ctDNA were stored at -80°C until use.

#### IFN-γ ELISpot assay

UCP-specific CD4^+^ T-cell responses were measured in all 60 evaluable patients by *ex vivo* IFN-γ ELISpot assay. Briefly, freshly isolated PBMCs were plated in a 96-well strip precoated ELISpot plate (Mabtech, Sweden) at 3.10^5^ cells/well in duplicate or triplicate with 5μg/ml of UCP2 and UCP4 (clinical grade, Provepharm, Marseille France) in X-Vivo 15 medium (Lonza, France) during 48h at 37°C, 5% CO_2_, without cytokine stimulation. Cells cultured with HIV-irrelevant derived peptide (JPT, Berlin, Germany) or phytohaemagglutinin (PHA) (1μg/ml) (Sigma-Aldrich, USA) were used as negative and positive controls respectively. The enzymatic revelation of ELISpot plate was performed according to manufacturer’s instructions by adding BCIP/NBT substrate (Mabtech). In selected experiments, 10 μg/ml of MHC Class I (Purified anti-human HLA-A,B,C Antibody, Clone W6/32, Biolegend) or MHC Class II (anti-human HLA-DR, Clone L243, Biolegend, anti-human HLA-DP, Clone B7/21, Leinco Technologies, anti-human HLA-DQ Clone 1a3, Leinco Technologies) blocking antibodies were added to peptide-stimulated wells.

Spots were counted using the CTL ImmunoSpot S6 analyzer (Cellular Technology Ltd, Germany) using ImmunoSpot software version 5.1. UCP-specific Th1 CD4^+^ T-cell counts were calculated as the mean spot counts in the UCP stimulated condition minus the mean spot counts in the control (HIV-derived peptide mixture). A response was considered positive if the number of UCP-specific IFNγ+ cells was twice the background and >10 specific T-cells.^67^ Data are presented as IFNγ spot number per 3.10^5^ cells after subtracting the background.

#### Assessment of epitope spreading

Patients with available PBMCs were assessed for epitope spreading measured by IFNγ ELISpot assay after a short *in vitro* stimulation as reported previously.^68^ Briefly, patients’ PBMCs (1.10^6^) were cultured in 48-well plate with 5μg/ml of non-vaccinated class II epitopes derived from the following tumor associated antigens (TAA): NY-ESO-1 (PepMix, JPT), mutated K-RAS (KRAS^G12V^ MTEYKLVVVGAVGVGKSALTIQLIQ), Mesothelin (Mesothelin_366-380_, Mesothelin_523-537_,^69^ JPT), KK-LC-1 (Class II peptides designed in our lab, ProImmune), and with HLA Class I-epitopes derived from hTERT (ProMix hTERT, ProImmune). Cells were cultured in RPMI supplemented with 5% human serum and recombinant IL-7 (5ng/ml; Preprotech) and IL-2 (20 UI/ml; Peprotech) were added at days 1 and 3 respectively. At day 6, stimulated cells were recovered and cultured in ELISpot plate (Mabtech) at 1.10^5^ cells/ triplicate well in presence of individual peptides derived from aforementioned TAA (5μg/ml) in X-Vivo 15 medium (Lonza, France) for 15 hours. Medium or PHA were used as negative and positive controls respectively. The ELISpot revelation and spot counting were performed according to manufacturer’s instructions as described above (Mabtech). Data are presented as IFNγ spot number per 10^5^ cells after subtracting the background. A positive epitope spreading was defined as presence of specific spots against at least one TAA occurred after vaccination. Patients with preexisting responses against TAA at baseline, which were maintained or expanded after vaccination was not considered as epitope spreading.

#### Intracellular cytokine secretion assay

*Ex vivo* intracellular cytokine secretion assay (ICS) was performed by flow cytometry using freshly isolated PBMCs collected at baseline and after 6 vaccinations (priming). Briefly, PBMCs (1.10^6^ cells/well) were stimulated in 5ml vial with UCP2 and UCP4 peptides (clinical grade) at 5 μg/ml) in 0.5ml of the X-vivo 15 medium (LONZA, USA). Two additional vials with 1.10^6^ cells were used for controls, unstimulated PBMCs and Phorbol Myristate Acetate (PMA, 1ng/ml) / ionomycine (500ng/ml) (Sigma-Aldrich, USA) for negative and positive controls respectively. Four hours later, protein transport inhibitor cocktail (BDGolgiPlug™, 1μg/ml^,^ BD Biosciences, USA) was added, and cells were incubated for a further 15h at 37°C, 5%CO_2_. After incubation, cells were stained with viability marker (1/1000^e^, Fixable viability dye, eBioscience, USA) and with surface antibodies: anti-CD3 Pacific Blue (clone UCHT1), anti-CD4 AlexaFluor 488 (clone RPA-T4), anti-CD8 PE-Cy7 (clone SK1) for 20min at 4°C. Cells were subsequently permeabilized and fixed with CytoFix/CytoPerm (BD Biosciences) and stained with intracellular antibodies: anti-IFNγ APC (clone 4S.B3), anti-TNFα Alexa Fluor 700 (clone IPM2), anti-IL-2 PE (clone MQ1-17H12) during 30min at 4°C. In some experiments, intracellular antibodies: anti-IL-4 PE (MP4-25D2) anti-IL-10 BV786 (clone JES3-9D7), anti-IL-17 Pacific Blue (BL168) were used. Cells were acquired on a CytoFlex™ cytometer (Beckman Coulter), and data were analyzed using Kaluza™ software version 2.1. The gating strategy is shown in Figure S7. Data are presented as percentage of cytokine positive CD4^+^ T cells after subtraction of background (unstimulated condition). ICS is positive if ≥2-fold increase between baseline and post-vaccination and more than 0.05%.

#### HLA-DR multimer staining and phenotypic analysis

UCP2 and UCP4-HLA-DR1 multimers were manufactured by the Peptide and Tetramer Core Facility (UNIL-CHUV, Epalinges, Switzerland). For multimer staining, we used an optimized peptide MHC class II multimer method.^70^ After thawing, PBMCs were resuspended in RPMI medium 10% human serum supplemented with LacNac (5mM) (Sigma-Aldrich) and incubated at 37°C for 2 hours. Cells were washed with PBS 1X, and resuspended in PBS 1X containing dasatinib (50nM) (Axon Medchem, Virginia, USA) and then incubated for 30 min at 37°C. After wash, PE and APC-conjugated UCP2-DR1 and UCP4-DR1 were added at a final concentration of 10 μg/mL for 45 min at room temperature (RT). Twenty minutes before the end of the multimer staining, 25 μL of the surface antibody cocktail was added directly without washing. After incubation, cells were washed and resuspended in PBS 1X and analyzed with the CytoFLEX Flow Cytometry Analyzer (Beckman Coulter). Data were analyzed using Kaluza™ software version 2.1. For phenotypic analysis the following markers were used. T cell polarization: anti-CD3 BV785 (Clone SK1), anti-CD4 BV510 (clone SK3), anti-CD8 PE-Cy7 (clone SK1), anti-IFNγ APC (clone 4S.B3), anti-CCR7 Brilliant Violet421 (clone G043H7), anti-CD45RA APC-Cy7 (clone HI100), anti-CXCR3 AlexaFluor488 (clone G025H27) and anti-CCR6 Brilliant Violet 650 (clone G034E3), anti-CXCR5 Brilliant Violet 605 (clone J252D4). Antibodies for specific T cell differentiation/activation: anti-CD3 AF700 (clone UCHT1), anti-CD4 AF488 (clone RPA-T4), anti-CCR7 BV650 (clone G043H7), anti-CD45RA APC-Cy7 (clone HI100), anti-CD39 PE Dazzle 594 (clone A1), ICOS BV605 (clone C398.4A), 4-1BB BV786 (clone 4B4-1). Antibodies for specific T cell cytotoxicity: anti-CD3 PE-Cy7 (clone UCHT1), anti-CD4 BV510 (clone SK3), anti-CD8 BV785 (clone SK1), anti-IFNγ APC (clone4S.B3), anti-GRZB PE-CF594 (clone GB11), anti-perforin AF488 (clone δG9), anti-TRAIL BV650 (clone RIK-2), anti-OX40 BV605 (clone Ber-ACT35).

For Tregs analysis, PBMCs were stained with anti-CD57 FITC (clone NC1), anti-CD28 PE (clone CD58.2), anti-CD127 (clone eBioDR5), anti-CD25 (clone M-A251), anti-CD3 (clone UCHT1), anti-CCR7 (clone G043H7), anti-CD4 (clone SK3), anti-CD45RA (clone HI100), and anti-CD8 (clone SK1) for 30 minutes at 4°. Cells were washed, fixed and permeabilized with BD Human FoxP3 Buffer according to the manufacturer's instructions (BD Biosciences). Cells were then stained with anti-FoxP3 APC (clone 259D) for 30 minutes at room temperature. After incubation, cells were washed with Stain Buffer (BD Biosciences) and analyzed with the CytoFLEX Flow Cytometry Analyzer (Beckman Coulter). Data were analyzed using Kaluza™ software version 2.1. All gating strategies are shown in Figure S7.

#### CD107a degranulation assay

Post vaccine PBMCs from patients were thawed and stimulated *in vitro* with pooled UCP2 and UCP4 at 5μg/ml for 6 days as described above. At day 7, stimulated cells were recovered and cocultured in 96-well plates with L-DR1 or L-DR4 target cells (kindly provided by Dr Maillère, CEA, France) loaded or not with UCP2 and UCP4 peptides at 1:1 Effector:Target ratio., in X-vivo 15 medium at 37°C, 5% C02. After 5 hours of incubation, protein transport inhibitor, GolgiStop at 1μg/ml (BD Biosciences) and anti-CD107a PE (clone H4A3) were added in the culture for 12 hours. The cells were then stained with anti-CD3 PE-Cy7 (clone UCHT1), anti-CD4 BV510 (clone SK3), anti-CD8 BV785 (clone SK1) and viability dye 7AAD (Beckman Coulter) for 30 minutes at 4°. Cells were washed and resuspended in PBS 1X and analyzed with CytoFLEX Flow Cytometry Analyzer (Beckman Coulter) and data were analyzed using Kaluza™ software version 2.1. The gating strategy is shown in Figure S6.

#### Antibody response

Anti-UCP antibody titers were measured by ELISA in plasma collected before and after vaccination. Coating was performed in 96-well NUNC plates (ThermoFisher, France) with 100μL of UCP2 + UCP4 peptides (1mg/ml), or with irrelevant MHC class II peptide derived from ovalbumin (5μg/ml) in PBS 1X. After overnight incubation at 4^0^C, wells were washed with PBS 1X 0.1% Tween 20 and then blocked 1h at RT with 5% nonfat milk in PBS with 0.1% Tween 20. Two dilutions of plasma in PBS 1X 1/125^e^ and 1/1000^e^ (100μL) were seeded in duplicate before being incubated 2h at RT. A positive control plasma was used in all assays to ensure assay compliance. After washing in PBS 0.1% Tween20, secondary antibody (Goat anti-human IgG HRP conjugate, Sigma Aldrich) was added and incubated 1h at RT before the addition of TMB substrate (Biolegend). The reaction was stopped with Sulfuric Acid (1N), and the OD was read on a plate reader (450nm, TECAN). To estimate the titer of UCP-specific IgG antibody, we used purified IgG1 immunoglobulin (R&D Systems), at serial dilution from 0.31 ng/mL to 20 ng/mL in PBS 1X. Anti-UCP IgG serum concentrations were extrapolated according to the polynomial expression derived from the standard curve. Data are presented as specific OD or IgG concentration (ng/ml) after subtracting the background. ELISA positivity was defined as OD or IgG two-fold increase in UCP coating-wells compared to the background (ovalbumin). If antibody against UCP was already detected at baseline, positive antibody response was considered if antibody titer or OD was at least two-fold increased after immunizations.

#### HLA genotyping and assessment of Patient harmonic-mean best rank (PHBR) score

HLA genotyping of 40 patients was performed on blood samples at inclusion. The HLA typing was performed by HLA laboratory of French Blood bank center (Besançon, France) using PCR sequence-specific oligonucleotides (NGS, Omixon, USA). The resolution of the major allele group was four digits. We used a presentation score defined by Marty et al.^42^^,^^71^ to represent the patient’s ability to present a residue given their distinct set of HLA alleles. First, we evaluated allele-based ranks for UCP2 and UCP4. Each allele-based rank was predicted using the NetMHCIIPan-4.0 tool, downloaded from the Center for Biological Sequence Analysis. NetMHCIIPan-4.0 takes a peptide and an MHC-II protein (HLA-DRB1, in our case) and returns binding affinity IC50 scores and corresponding allele-based ranks (BR). Second, the PHBR (Patient Harmonic-mean Best Rank) score was assigned as the harmonic mean of the best residue presentation scores for HLADRB1 alleles and calculated as follow:

$$\text{PHBR}=\frac{2}{\sum_{i=1}^{2}\;\frac{1}{BRi}}$$

A lower patient presentation score indicates that the patient’s HLA-alleles are more likely to present a peptide on the cell surface.

#### Cytokine measurement

For some experiments, IL-4, IL-5, IL-13, IL-9, IL-17, and IL-10 cytokines were measured in the supernatant of patients’ PBMCs stimulated during 48h with UCP peptides (5μg/ml each).

In other experiments, TNF-α, IL-6, IL-1β, IL-8, IL-10 were measured in the plasma of patients. These cytokines were measured using Cytometric Bead Array (CBA) Flex Set (BD Biosciences, France) according to manufacturers’ instruction. Data were analyzed using software FCAP array version 3 (BD Biosciences, France).

#### UCP-specific CD4^+^ T cell sorting for scRNAseq and TCR sequencing

UCP-specific CD4^+^ T cells were sorted by using IFNγ Secretion Assay- Detection kit (Miltenyi Biotec, Germany). PBMC from selected patients were thawed and *in vitro* stimulated during 6 days with UCP2 and UCP4 peptides (5μg/ml) as described above in Epitope Spreading assessment. On day 7, cells were plated at 5.10^6^ cells/mL and stimulated for 4 h with UCP2 and UCP4 peptides (5μg/ml) at 37°C. According, the manufacturer’s instructions (ref 130-090-433), cells were labelled with IFNγ capture reagent and cultured 45min at 37°C to allow IFNγ secretion. Cells were next labeled with IFNγ detection antibody and with following antibodies: anti-CD3 APC (clone UCHT1), anti-CD4 BV421 (clone RPA-T4) and viability die eFluor506 (ThermoFisher, France). IFNγ secreting cells were sorted on MACSQuant Tyto (Miltenyi Biotec, Germany) using MACSQuant Tyto Cartridges HS according to a gating strategy excluding dead cells and selecting positive IFNγ+CD4^+^ T cells and negative fractions (IFNγ-CD4^+^) as control.

#### Single cell RNA sequencing (scRNA-seq)

IFNγ-CD4^+^ T cells were encapsulated using the 10x Genomics Chromium Controller and the Chromium Next GEM Single cells 5′ Library Kit v2 (PN-1000265; 10x Genomics, USA) (3500 cells of patient #23, 8600 cells of patient #5, 8500 cells of pooled patients #20 and #37, and 32000 cells for bulk CD4^+^ from patient #23’s baseline were encapsulated). The integrity of the cDNAs was evaluated using Agilent Bioanalyzer High Sensitivity Chip (Agilent Technology, USA). To prepare the final libraries using dual index kit TT set A (PN-1000215, 10x Genomics), amplified cDNA was enzymatically fragmented, and size selected using SPRIselect magnetic beads (B23318, Beckman Coulter) followed by ligation of Illumina sequencing adapters. The quality of the final library was assessed using an Agilent Bioanalyzer High Sensitivity chip. Sequencing was performed on an Illumina Novaseq 6000 device. Libraries were sequenced with 150-bp paired-end reads.

#### scRNA-seq data analysis

The raw data files were demultiplexed into FASTQ file by cellranger mkfastq. v.7.1.0. Then reads were aligned against GRCh38 human reference genome 2020-A provided by 10x Genomics based on GENCODE v32/Ensembl98. Using the Read10X function of the Seurat v.4.9.9 package of R v.4.2.2, we loaded expression data from the cellranger count pipeline into R and then created Seurat Object by CreateSeuratObject, with the selection criterion being cells expressing at least 200 genes expressed in more than 3 cells. Cells with 300-5000 unique genes and < 20% mitochondrial reads were retained for downstream analyses. To filter out doublet and multiplet cells, DoubletFinder v. 2.0.4 was used. Count matrices were merged to a single Seurat object. Cell cycle phases assignation was done by using Seurat's CellCycleScoring function. Log normalized counts were used to identify 2000 most variably expressed genes using the FindVariableFeatures function with the variance stabilizing transformation. Integration was performed using respectively SelectIntegrationFeatures, PrepSCTIntegration and FindIntegrationAnchors of Seurat. Principal component (PC) analysis was performed, and the first 10 PCs were selected for UMAP dimensionality reduction. To cluster cells, Seurat's FindNeighbors method was used to determine the biologically closest cells, before grouping them together by using Seurat's FindClusters function with a resolution of 0.3. The cell anotation was done by identifying the marker genes of these clusters using Seurat's FindAllMarkers method with the parameters only.pos = TRUE, min.pct = 0.25 and logfc.threshold = 0.25, and then studying the expression of the T cell differentiation, function and exhaustion markers.

To evaluate polarization gene signatures, UCell v 2.6.2 scoring was applied. The signatures were defined based on CellMarker2.0. The cell trajectory analysis from our Seurat object was done by using the package monocle3 v 1.3.7.

Inferring cell-cell communication from our seurat object was done by using the package cellchat v. 1.5.0.^72^ Cell subtypes with less than 10 cells are filtered out.

#### TCR-β sequencing and analysis

TCR-β analysis was performed on UCP-specific T cells from vaccinated patients sorted by using IFNγ Secretion Assay- Detection kit, as described above. Negative fraction of each patient were used as control.

IFNγ+ and IFNγ- CD4+T cells were lysed in 300μL Lysis/Binding Buffer for Dynabeads™ mRNA Purification Kits and stored at -80°C (A33562, Invitrogen). Bulk TCR sequencing analyses were performed as described previously.^73^ Briefly, mRNA was isolated and amplified by *in vitro* transcription. 5’ adapter was added by multiplex reverse transcription and TCRs were amplified using one primer in the adapter and one in the constant region. Libraries were sequenced on Illumina instrument and TCR sequences processed using an ad hoc Perl script.

Divers indexes such are richness, Shannon Entropy and clonality were used to analyze the repertoire. Richness refers to the number of unique clonotypes identified in the repertoire. The Shannon Entropy was calculated as follow: $-\sum_{i=1}^{n}F_{i}*\log\;2\left(F_{i}\right)$.

Where n is the total number of clonotype and F the clonotype frequency. Clonality, refers to 1-Pielou index, was calculated as follow: $1-\left(\frac{-\sum_{i=1}^{n}F_{i}*\log\;10\left(F_{i}\right)}{\log\;10\left(n\right)}\right)$.

Where n is the total number of clonotypes and F the clonotype frequency.

MiXCR upstream analysis ouput were used as input for the immunarch v.0.9 package. The distribution of clones was done by repExplore (method = "volume"). Clonality analysis was assessed using repClonality (method = "homeo") function and diversity evaluation was done by repDiversity function (method = "d50"). The trackClonotypes function (col = ‘aa+v’) was used to track clonotypes.

### Quantification and statistical analysis

Overall Survival was calculated as the time from inclusion to the date of death and estimated using the Kaplan-Meier method, described using median or rate at specific time points with 95% Confidence Intervals (CI) using R software, package Survimmer. Alive patients were censored at last date known to be alive. Follow-up duration was calculated using a reverse Kaplan-Meier estimation. The log-rank test was used to compare survival curves. Cox proportional hazard models were performed to estimate hazard ratio (HR) and 95% CI for factors associated with OS. Quantitative variables are described as median and range. The Shapiro-Wilk test was used to test the normality of the data. Multiple groups comparison was performed using the Kruskal-Wallis tests and two groups comparisons were done with the Wilcoxon-Mann-Whitney test. Proportions were compared using the Chi2-test or Fisher when appropriate. The ggwithinstats function of the ggstatsplot v.0.11.1 package was used to visualize statistical results. Considering the Post hoc nature of the analyses and the absence of consideration for multiple tests correction p-value were provided in an exploratory purpose. P values lower than 0.05 were considered as statistically significant (∗p<0.05, ∗∗p<0.01, ∗∗∗p<0.001). Statistical analyses were performed using R software version 4.2.3 (http://www.R-project.org/), and graphs were drawn using GraphPad Prism version 10.0.2 or R using ggplot2 v3.5.0. Calculations were performed using high-performance computing resources from the “Mésocentre de calcul de Franche-Comté”.

### Additional resources

The full protocol is accessible on ClinicalTrials.gov, NCT02818426.

## Supplemental information

Document S1. Figures S1–S7 and Tables S1–S5

Document S2. Article plus supplemental information
