# Supplementary material for: UCPVax, a CD4 helper peptide vaccine, induces polyfunctional Th1 cells, antibody response, and epitope spreading to improve antitumor immunity
Source: Cell Rep Med. 2025 Jun 20;6(7):102196. doi: 10.1016/j.xcrm.2025.102196 (PMC12281370; doi:10.1016/j.xcrm.2025.102196)
Supplement: Document S1. Figures S1–S7 and Tables S1–S5 [file mmc1.pdf]

Cell Reports Medicine, Volume *6*

Supplemental information

UCPVax, a CD4 helper peptide vaccine, induces polyfunctional Th1 cells, antibody response, and epitope spreading to improve antitumor immunity

Caroline Laheurte, Laura Boullerot, Babacar Ndao, Marine Malfroy, Lise Queiroz, Phillippe Guillaume, Romain Loyon, Evan Seffar, Eleonore Gravelin, Adeline Renaudin, Marion Jacquin, Aurélia Meurisse, Dewi Vernerey, François Ghiringhelli, Yann Godet, Raphael Genolet, Camilla Jandus, Christophe Borg, and Olivier Adotévi

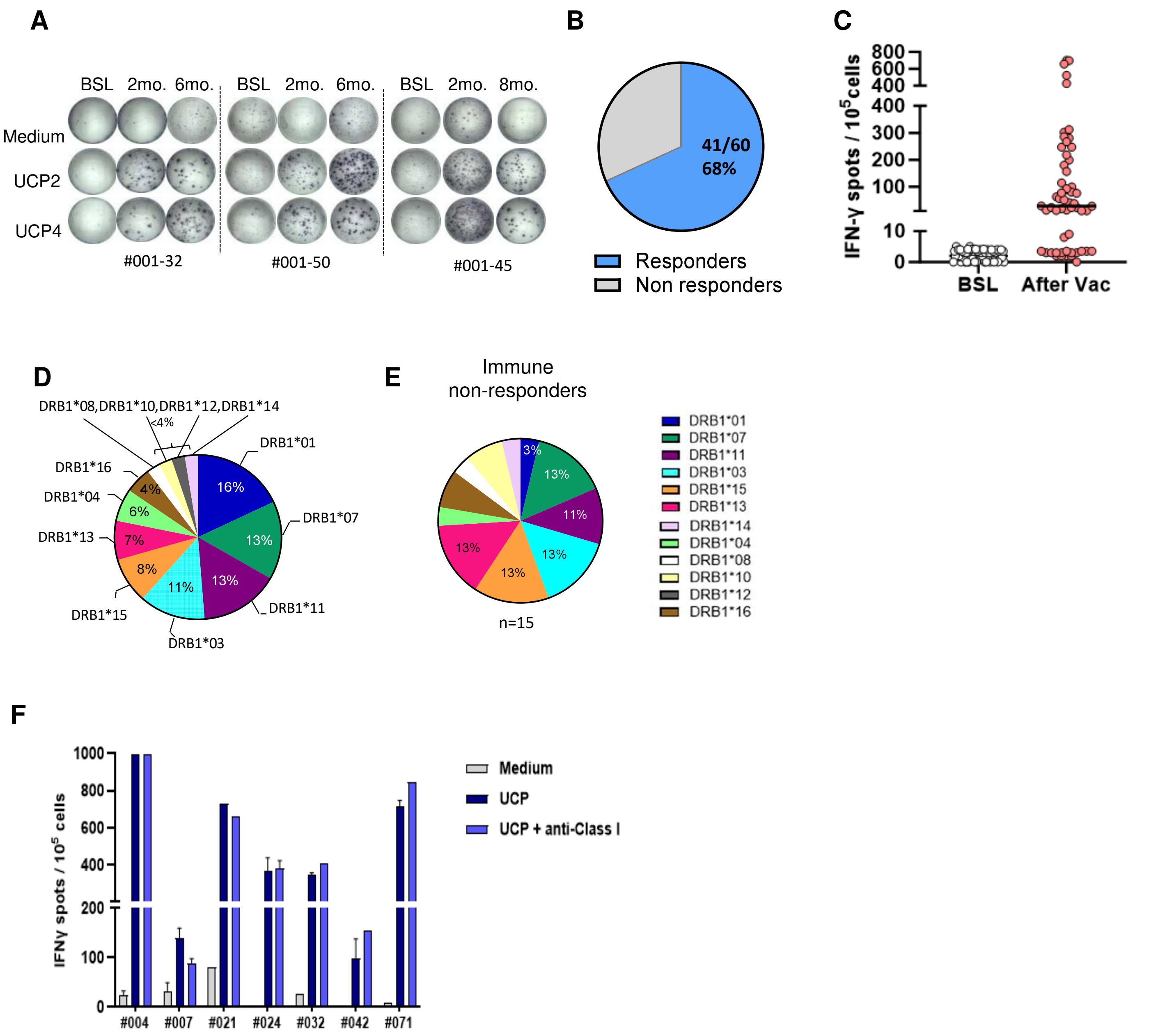

Supplementary Figure 1. Overall immunogenicity of UCPVax by *ex vivo* IFNγ ELISpot . Related to Figure 1
(A) IFNγ ELISpot wells of UCP2- and UCP4- specific CD4+ T cells from three representative patients at baseline (BSL) and at different time after vaccination.
(B) Overall immunogenicity (frequency of UCP2/UCP4-specific immune responses at any time after vaccine).
(C) Magnitude of UCP-specific CD4+ T cell responses pre and post-vaccine.
(D) HLA-DR-B1 alleles frequencies distribution in all patients (n=60).
(E) HLA-DR-B1 alleles frequencies distribution in immune non-responders to UCPVax (n=15)
(F) Representative examples of UCP specific CD4+ T cell responses with the addition or not of anti-class I antibody in the ELISpot assay (n=7)

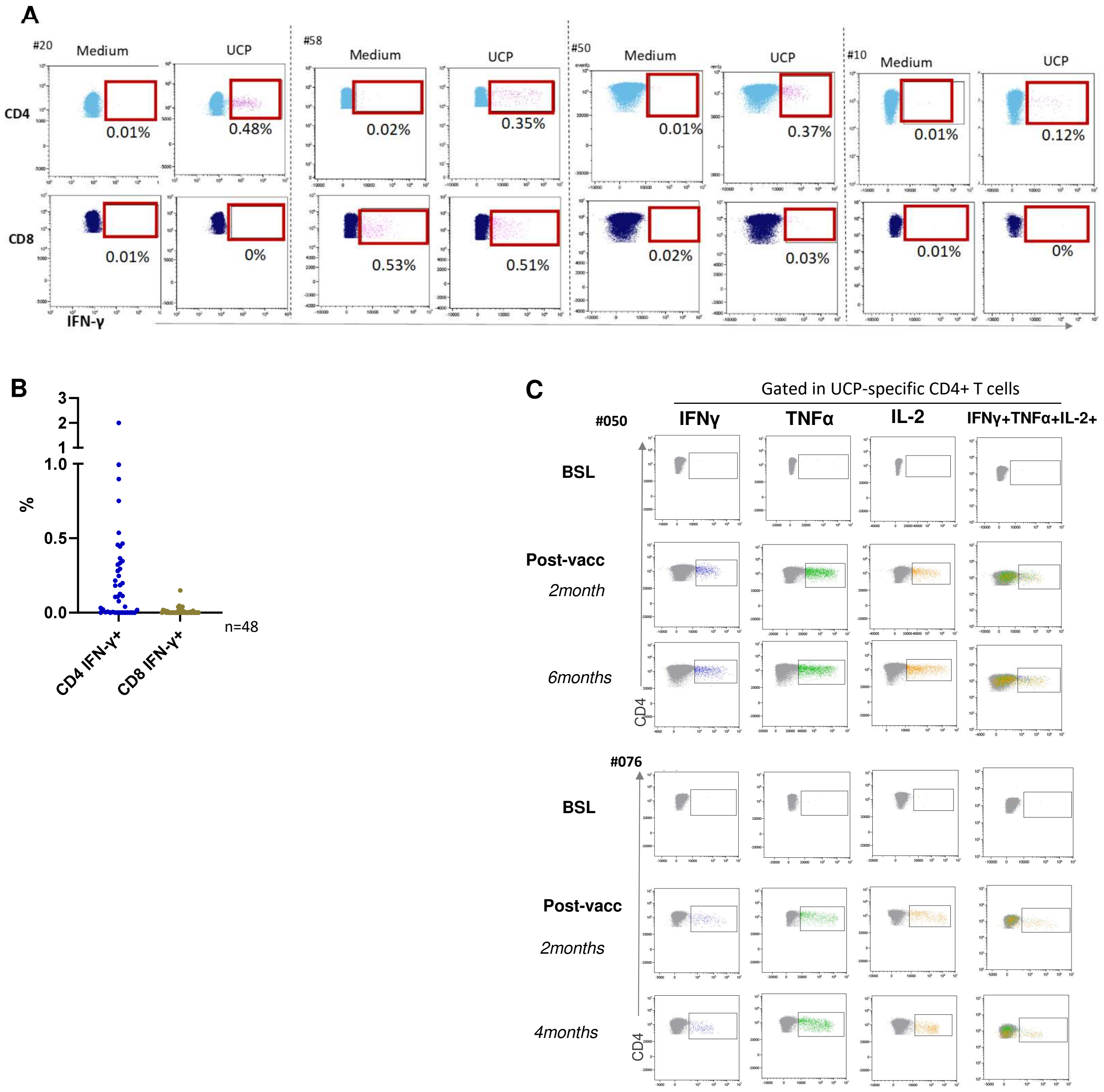

Supplementary Figure 2. UCPVax stimulates CD4+ but not CD8+ T cell responses. Related to Figure 2
(A) Representative examples of flow cytometry dot plots showing IFNγ production in CD4+ T cells and CD8+T cells after vaccination by *ex vivo* ICS.
(B) Percentage of IFNγ in CD4 and CD8 T cells after ex vivo ICS in post-vaccination (n= 48 patients)
(C) Representative examples of flow cytometry dot plots showing UCP-specific CD4+ T cells producing IFNγ, IL-2, and TNFα at individual secretion and simultaneously along vaccination.

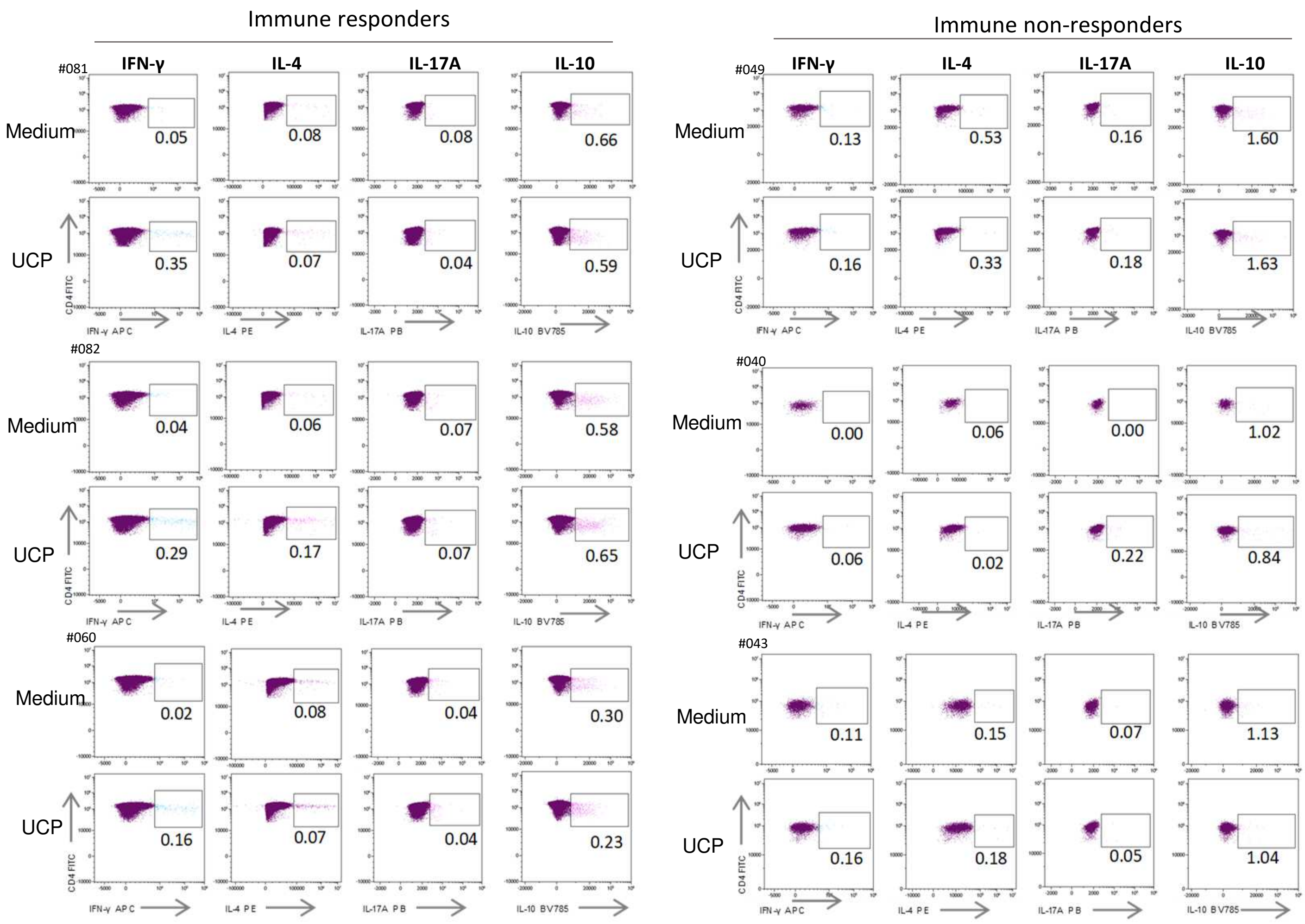

Supplementary Figure 3. Assessment of Th2, Th17 or Treg polarization after UCPVax vaccination Related to Figure 2. Representative ICS dot plots of IL-4, IL-17A, and IL-10 production in CD4+T cells of immune responder (n=3) and non-responder (n=3) patients after vaccination

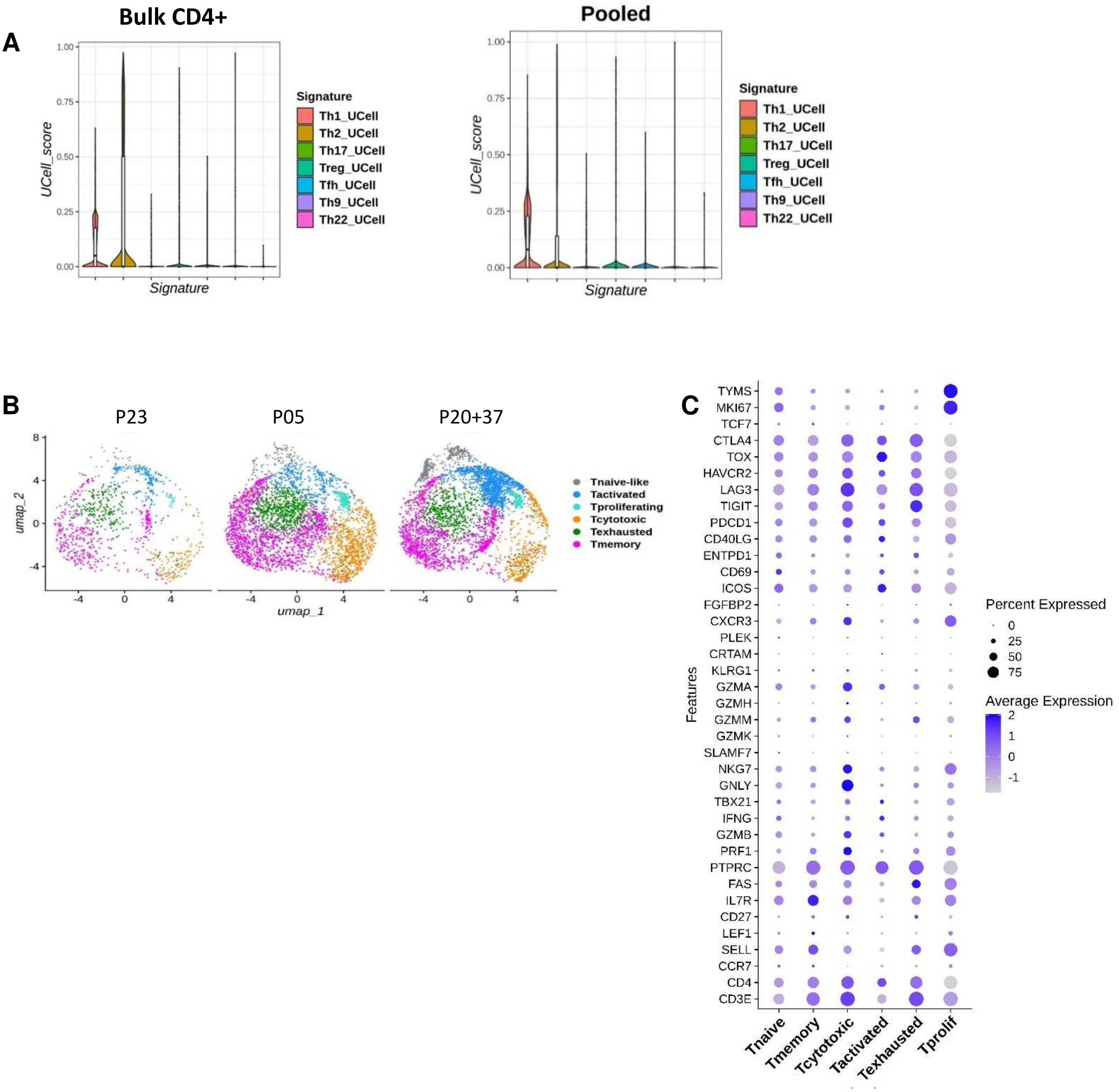

Supplementary Figure 4. Single cells transcriptomic analysis of UCPVax-expanded CD4+ T cells. Related to Figure 3.
(A) UCell scoring of T helper polarization gene signatures in single-cell dataset from bulk CD4 T cells (negative fraction) and from pooled post vaccine specific samples from all the four patients (#23, #05 #20, #37)
(B) UMAP analysis of UCP-specific CD4 T cells differentiation clusters from individuals' patients after vaccinations, (colors corresponding to the cell cluster identified)
(C) Average gene expression of activation, cycling, polarization, and cytolytic markers of UCP-specific CD4 T cells differentiation clusters identified in the UMAP in Figure 3D
Size of the dot indicates the percent expression among cells and the color represents the average gene expression.

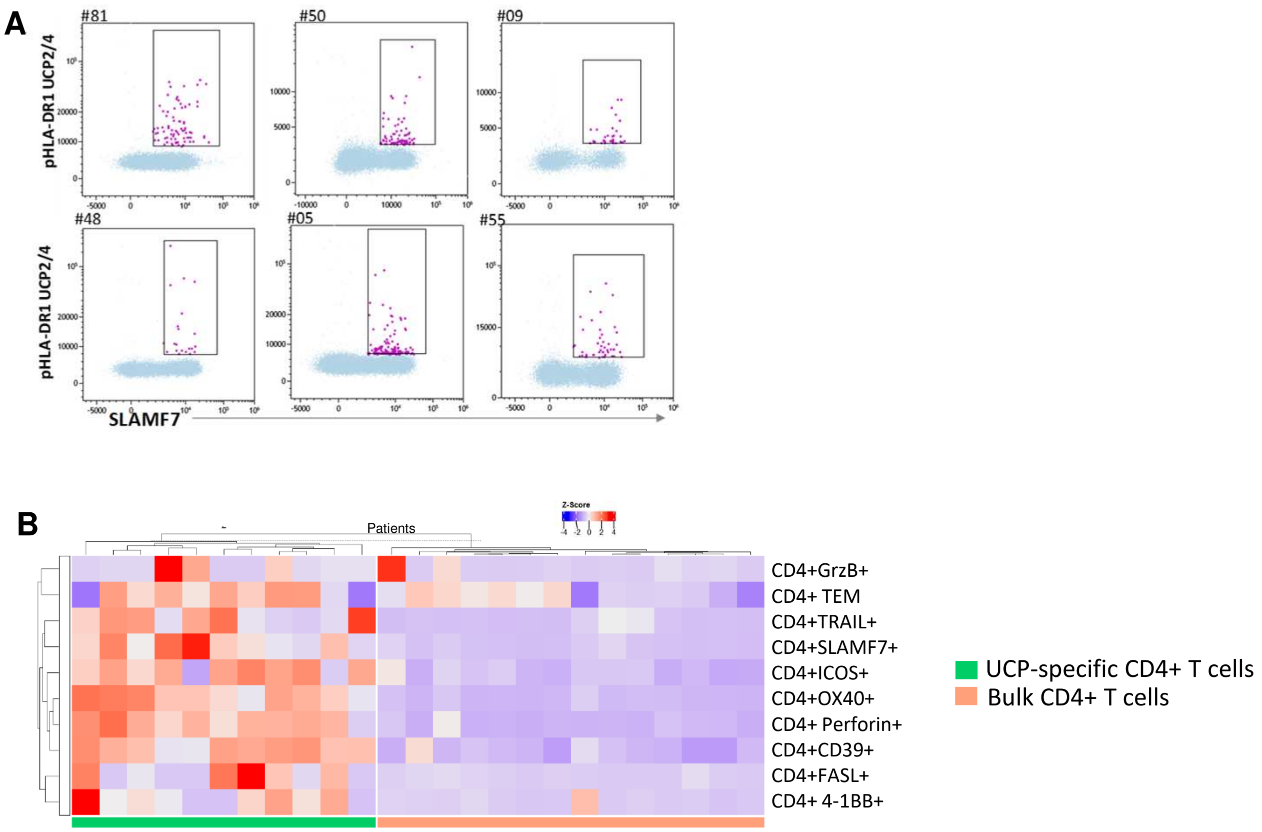

Supplementary Figure 5. Activation, differentiation, and cytotoxic markers in vaccine specific CD4+ T cells. Related to Figures 3 and 4

(A) Flow cytometry dot plots showing SLAMF7 expression on pHLA-DR1 multimer in 6 vaccinated patients

(B) Unsupervised heatmap showing cytotoxic and activation markers expressed on bulk versus UCP-specific CD4+ T cell in 10 vaccinated patients.

A

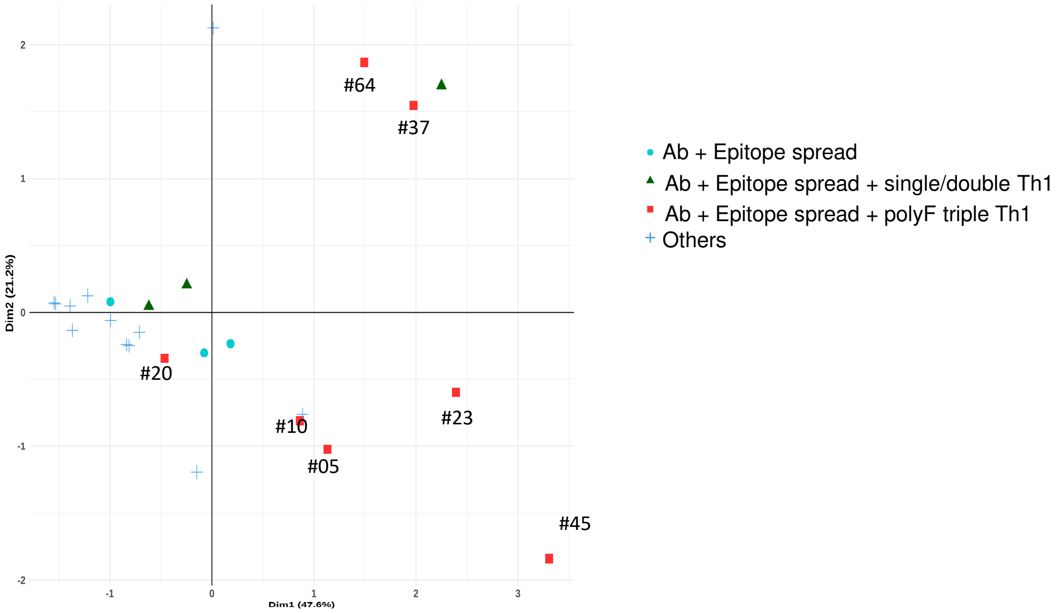

B

Patients	HLA-DRB1 allele	% polyF triple+ Th1	Epitope Spread response	Ab response IgG titer (ng/ml)	median OS (months)
001-064	DRB1*01:01	0.059	POS	226	19.3
001-037	DRB1*01:01	0.068	POS	4610	26.7
001-045*	DRB1*04:01	0.323	POS	4981	11.1
001-023*	DRB1*13:01	0.185	POS	5102	53.0
001-005	DRB1*01:01	0.166	POS	6208	5.9
001-010	DRB1*11:01	0.166	POS	6796	22.0
001-020	DRB1*04:05	0.051	POS	5007	17.8

*Complete Response (CR), Partial Response (PR)

Supplementary Figure 6. Patients with vaccine-induced immune triad. Related to Figure 6.
(A) Unsupervised principal component analysis (PCA) showing individual patients according to the group of immune response
(B) Table showing median OS of each of the 7 patients cumulating the three main type of anti-tumor immune responses promoted by UCPVax (polyF triple +Th1, epitope spreading, antibody response).

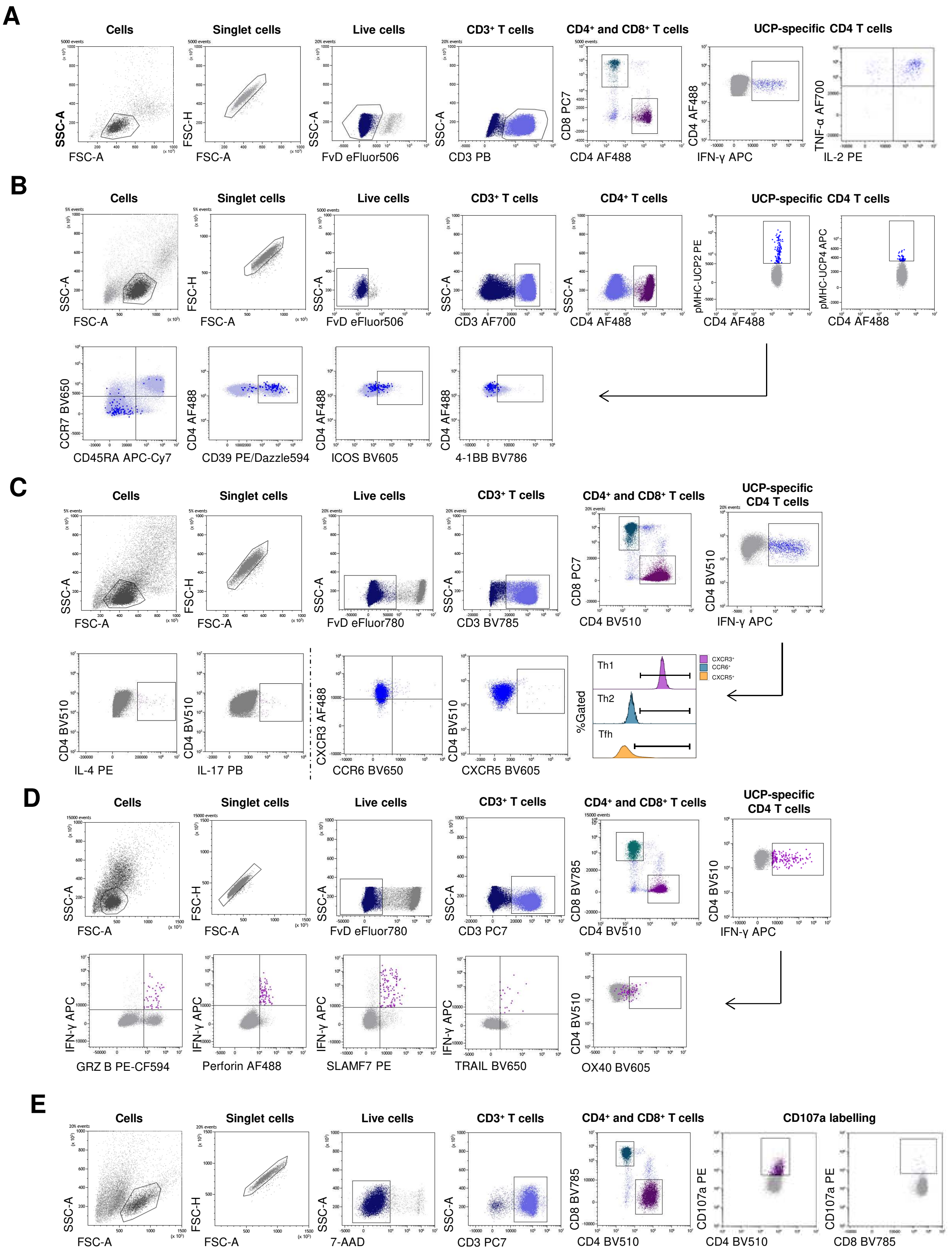

Supplementary Figure 7: Gating strategy of flow cytometry analysis. Related to Figures 2,3 and 4
(A) ICS assays of IFNγ, TNFα, IL2. (B) pMHC class II UCP2 and UCP4 multimer and activation markers. (C) UCP-specific Th1 phenotypic characterization. (D) UCP-specific cytolytic phenotypic characterization. (E) CD107a degranulation assay.

**Baseline characteristics**	Number of patients (%)
Median [range] age, years (n=60)	67.1 [62.8-72.2]
Gender (men/women)	41 (68.3)/19 (31.7)
Tabac (n=60)	
Never smoker	5 (8.3%)
Current or former smoker	55 (91.7)
ECOG PS, *n* (%)	
0	19 (31.7%)
1	41 (68.3%)
Stages IV	60 (100%)
Histology	
Adenocarcinoma	35 (58.3%)
Squamous cell	22 (36.7%)
Others	3 (5%)
PD-L1 expression (n=37)	17 (45.9%)
Mutations	
EGFR (n=35)	2 (5.7%)
KRAS (n=37)	14 (37.8%)
BRAF (n=34)	2 (5.9%)
HER2 (n=30)	0 (0.0%)
ALK translocated (n=35)	0 (0.0%)
ROS1 fusion (n=21)	0 (0.0%)
Previous therapy	
Chemotherapy	60 (100%)
Immunotherapy	60 (100%)
Targeted Therapy	2 (3.3%)

Supplementary Table S1. **Main baseline patient's clinical characteristics.**
Related to Figure 1

#Patient	HLA typing		UCP2-specific IFNγ spots	PHBR score UCP2	UCP4-specific IFNγ spots	PHBR score UCP4
	HLA-DRB1 Allele 1	HLA-DRB1 Allele 2				
#002	-	-	23	-	0	-
#003	DRB1*07:01	DRB1*14:01/14:54	99	2.01	0	17.34
#004	DRB1*11:01	-	698	10.55	698	4.23
#005	DRB1*01:01	DRB1*03:01	114	0.54	10	0.56
#006	DRB1*03:01	-	0	59.27	0	55.00
#007	DRB1*01:01	-	10	0.27	8	0.28
#009	DRB1*01:02	DRB1*03:01	29	2.39	0	3.28
#010	DRB1*11:01	DRB1*12:01	199	6.44	0	15.49
#013	DRB1*11:01	DRB1*14:02	0	10.72	0	15.38
#017	DRB1*10:01	DRB1*15:01	0	3.97	0	2.44
#020	DRB1*04:05	DRB1*11:03	63	15.59	57	17.06
#021	DRB1*11:04	DRB1*12:01P	13	7.81	0	15.52
#022	DRB1*01:01	DRB1*03:01	700	0.54	133	0.56
#023	DRB1*13:01	-	247	25.53	63	28.39
#024	DRB1*01:02	DRB1*14:54	14	2.31	0	3.19
#028	DRB1*07:01	DRB1*11:01	88	1.91	27	6.31
#029	-	-	10	-	11	-
#030	Missing data	-	41	-	36	-
#032	DRB1*07:01	DRB1*16:01	43	0.56	56	2.16
#036	DRB1*08:01	DRB1*15:01	218	9.26	76	12.08
#037	DRB1*01:01	DRB1*07:01	698	0.43	698	0.55
#039	DRB1*01:01	DRB1*07:01	180	0.43	92	0.55
#042	DRB1*03:147	DRB1*11:04	19	NA	9	NA
#045	DRB1*04:01	DRB1*11:01	151	11.21	281	6.07
#049	DRB1*13:01	DRB1*16:01	0	0.75	0	2.26
#050	DRB1*01:02	DRB1*11:01	161	2.19	302	2.42
#051	DRB1*11:01	DRB1*16:01	80	0.73	68	1.85
#052	DRB1*11:01	DRB1*13:03	157	14.25	16	7.58
#055	DRB1*01:01	DRB1*07:01	312	0.43	312	0.55
#057	DRB1*15:01	-	0	6.83	0	10.95
#060	DRB1*07:01	-	286	1.05	0	12.44
#061	DRB1*03:01P	DRB1*10:01	0	5.49	0	2.67
#062	DRB1*04:01	DRB1*08:01	0	13.04	0	11.94
#064	DRB1*01:01	DRB1*05:01	659	NA	306	NA
#065	DRB1*03:01	DRB1*15:01	23	12.25	13	18.26
#075	DRB1*07:01	DRB1*14:01P	678	2.00	48	17.34
#076	DRB1*04:04	DRB1*04:05	55	16.44	247	16.18
#077	DRB1*04:02	DRB1*16:01	0	0.75	0	2.12
#078	DRB1*13:01	DRB1*15:01	74	10.78	0	15.80
#079	DRB1*13:02	DRB1*13:05	267	12.92	117	7.25
#080	DRB1*07:01	DRB1*08:01P	0	1.96	0	12.93
#081	DRB1*01:01P	DRB1*07:01	522	0.43	522	0.55
#082	DRB1*07:01	DRB1*15:02	429	1.36	429	9.59
#040	DRB1*07:01	DRB1*11:01	0	1.91	0	6.31
#043	DRB1*04:05	-	0	11.35	0	21.17
#056	DRB1*03:01P	DRB1*16:01	0	0.76	0	2.31
#058	DRB1*01:01	DRB1*11:03	93	0.53	93	0.55
#014	DRB1*03:01	DRB1*07:01	0	2.06	0	20.29
#016	Missing data	-	23	-	0	-
#018	Missing data	-	0	-	0	-
#041	Missing data	-	0		0	
#048	DRB1*07:01	DRB1*01:03	0	0.53	0	0.20
#063	DRB1*04:01	DRB1*12:01P	12	6.67	0	13.15
#067	DRB1*07:01	DRB1*13:03	0	2.00	0	18.56
#069	DRB1*03:01P	DRB1*01:03	10	0.70	0	0.20
#072	DRB1*13:02	DRB1*15:02	0	9.69	0	15.29
#074	DRB1*08:01P	DRB1*11:01P	0	12.16	0	6.44
#044	DRB1*01:01	DRB1*07:01	76	0.43	37	0.55
#047	DRB1*13:02	DRB1*15:01	0	9.69	0	15.29
#071	DRB1*15:01	-	933	6.83	933	10.95

NA, not applicable

Supplementary Table S2: **HLA-DR alleles, *ex vivo* IFNγ ELISpot and PHBR score.**
Related to Figure 1

#Patient	Immune status ELISpot anti-UCP	IL-4 (pg/ml)		IL-5 (pg/ml)		IL-13 (pg/ml)		IL-9 (pg/ml)		IL-17A (pg/ml)		IL-10 (pg/ml)	
		Medium	UCP2&4	Medium	UCP2&4	Medium	UCP2&4	Medium	UCP2&4	Medium	UCP2&4	Medium	UCP2&4
#005	Positif	0.00	0.77	0.00	0.00			0.00	0.00	0.00	0.00	0.00	3.24
#006	Negatif			0.00	0.00	0.00	0.00	0.00	0.00	0.00	0.00	9.98	6,00
#010	Positif	0.00	0.03	0.00	0.00			0.00	0.00	0.00	0.00	0.00	0.91
#017	Negatif			0.00	0.00	0.00	0.46	0.00	0.00	0.00	0.00	212.00	61.15
#020	Positif	0.00	0.64	0.00	0.00			0.00	0.00	0.00	0.00	0.00	2.32
#029	Positif			0.00	0.00	0.10	0.00	0.00	0.00	0.02	0.30	1.07	1.95
#032	Positif	0.00	0.3	0.00	0.00			0.00	0.00	0.00	0.00	0.00	2.41
#037	Positif			0.00	0.00	0.00	0.3	0.00	0.00	0.00	0.00	0.95	0.7
#039	Positif	0.00	0.27	0.00	0.00			0.00	0.00	0.00	0.00	0.00	0.81
#045	Positif	0.00	0.23	0.00	0.00			0.00	7.575	0.00	0.00	0.00	3.43
#049	Negatif			0.00	0.00	0.00	0.00	0.00	0.00	0.00	0.00	0.00	0,00
#050	Positif	0.00	0.41	0.00	0.00			0.00	0.00	0.00	0.00	0.00	1.66
#051	Positif	0.00	0.59	0.00	0.00			0.00	16.97	0.00	0.00	0.00	15.41
#055	Positif	0.00	0.88	0.00	0.00			0.00	148.9	0.00	0.00	0.00	39.77
#057	Negatif	0.00	0.36	0.00	0.00			0.00	0.00	0.00	0.00	0.00	1.115
#060	Positif	0.00	0.36	0.00	0.00			0.00	0.00	0.00	0.00	0.00	6.74
#062	Negatif	0.00	0.93	0.00	0.00			0.00	0.00	0.00	0.00	0.00	1.63
#064	Positif	0.00	0.94	0.00	0.00			0.00	0.00	0.00	0.00	0.00	3.96
#075	Positif	0.00	0.31	0.00	0.00			0.00	12.06	0.00	0.00	0.00	2.35
#076	Positif			0.00	20.89	1.09	105.05	0.10	5.97	0.09	2,76	3.51	13.40
#079	Positif	0.00	0.99	0.00	0.00					0.00	3.25	0.00	11.01
#040	Negatif			0.00	0.00	0.00	0.00	0.00	0.00	0.00	0.00	0.10	0.15
#043	Negatif			0.00	0.00	0.00	0.00	0.00	0.00	0.00	0.06	4.17	1.33
#056	Negatif	0.00	0.33	0.00	0.00					0.00	0.00	0.00	1.87
#058	Positif			0.00	0.00	0.00	0.00	0.00	0.00	0.00	0.00	1.29	1.27
#014	Negatif	0.00	0.075	0.00	0.00					0.00	0.00	0.00	1.38
#018	Negatif			0.00	0.00	0.00	0.00	0.00	0.00	0.00	0.00	3.75	3.6
#063	Positif			0.00	0.00	0.00	0.00	0.00	0.00	0.00	0.00	0.39	0.24
#067	Negatif			0.00	0.00	0.00	0.00	0.00	0.00	0.00	0.01	0.49	0.08
#069	Positif	0.00	0.53	0.00	0.00					0.00	0.00	0.00	1.38
#074	Negatif	0.00	0.19	0.00	0.00					0.00	0.00	0.00	1.20
#044	Positif	0.00	0.19	0.00	0.00					0.00	0.00	0.00	1.44
#047	Negatif			0.00	0.00	0.00	0.00	0.00	0.00	0.00	0.00	0.00	0.10

Supplementary Table S3: Cytokines production by vaccine-specific CD4 T cells

Related to Figure 2

Cytokines measured by CBA in the supernatant of UCP2 /UCP4 -stimulated PBMCs (n= 48 patients)

Cox regression analyses	**Multivariate**		
	**HR**[a]	**95%CI** [b]	***P* value**
**PolyF triple+ Th1**			
Negative (n=24)			
Positive (n=10)	0.30	0.11-0.83	**0.02**
**Epitope Spreading**			
Negative (n=20)			
Positive (n=15)	0.42	0.16-1.09	*0.08*
**UCP-specific Antibody**			
Negative (n=15)			
Positive (n=19)	1.64	0.54-4.96	0.38

a : Hazard ratio
b : Confidence Intervals

Supplementary Table S4 : **Multivariate cox regression analysis.**
Related to Figure 6

	PolyF Th1			Ab response			Epitope Spreading		
	Others	Triple+ Th1		NEG	POS		NEG	POS	
	n=30	n=18	*P value*	n=19	n=31	*P value*	n=26	n=15	*P value*
**Age, years**<br>Mean (std)	67.7 (7.4)	65.5 (7.0)	ns	69.1 (9.4)	64.8 (6.9)	ns	65.8 (8.6)	65.9 (7.4)	ns
**Gender,** No. (%)									
men	18 (60%)	14 (77.8%)	ns	12 (63.1%)	21 (67.8%)	ns	18 (69%)	11 (73.3%)	ns
women	12 (40%)	4 (22.2%)		7 (36.8%)	10 (32.2%)		8 (31%)	4 (26.6%)	
**Histology,** No. (%)									
Adenocarcinoma	17 (56.7%)	11 (61.1%)	0.42	14 (73.6%)	16 (51.6%)	0.19	14 (53.8%)	9 (60%)	0.36
Squamous cell	13 (43.3)	5 (27.8%)		5 (26.3%)	13 (41.9%)		12 (46.2%)	4 (27%)	
Others	0	2 (11.1%)			2 (6.4%)			2 (13.3%)	
**Mutations KRAS,** No. (%)									
WT	11 (73.3)	6 (46.1)	0.14	9 (64.3%)	9 (52.9%)	0.52	11 (78%)	6 (60%)	0.32
Mutated	4 (26.7)	7 (53.8)		5 (35.7%)	8 (47.0)		3 (22%)	4 (40%)	
**PD-L1 expression,** No. (%)									
No	9 (60%)	5 (41.7)	ns	6 (54.5%)	10 (52.6%)	ns	6 (37%)	3 (60%)	ns
Yes	6 (40%)	7 (58.3)		5 (45.4%)	9 (47.4%)		10 (63%)	2 (40%)	
**Duration immunotherapy lines**<br>Months, Mean (std)	6.2 (7.8)	10.6 (11.8)	0.14	5.1 (4.7)	8.1 (9.7)	0.35	5.1 (6.2)	8.9 (11.1)	0.17
**Total Lymphocytes count**<br>Mean (Std)	1453.5 (937.1)	1316.7 (446)	ns	1320 (1000)	1397 (597)	ns	1380.4 (893)	1358.2 (562)	ns
**NLR,** Mean (Std)	4.6 (3.1)	5.7 (4)	ns	5.6 (3.3)	4.7 (3.2)	ns	5.4 (3.0)	4.2 (2.2)	ns
**CD4 % ,** Mean (Std)	55.4 (14.9)	46.0 (19.2)	ns	50.7 (13.5)	49.2 (18.8)	ns	53.5 (14.9)	53.9 (15.4)	ns
**CD4 count,** Mean (Std)	835.2 (664.3)	597.9 (287.5)	ns	712.3 (739.7)	709.4 (454.4)	ns	803.3 (688.2)	803.5 (532.9)	ns
**LDH,** Mean (Std)	314.8 (135)	249.7 (59.6)	ns	310.5 (134.9)	255.2 (80.2)	ns	309.5 (126.0)	268.6 (119.5)	ns
**Inflammatory cytokines,** Mean (Std)									
**IL-1β**	0.29 (0.48)	0.82 (2.6)	ns	0.33 (0.54)	0.54 (1.91)	ns	0.20 (0.22)	0.80 (2.5)	0.62
**IL-6**	12.2 (13.8)	4.3 (4.5)	**0.02**	13.8 (15.1)	6.1 (7.3)	**0.05**	13.3 (14.2)	5.6 (5.9)	**0.07**
**IL-8**	27.1 (33.1)	10.0 (9.3)	**0.09**	28.8 (35.4)	12.7 (16.6)	**0.03**	21.5 (20.1)	16.5 (32.8)	**0.04**

ns : non significant ; No. : number ; Std : standard deviation
NLR: Neutrophil to lymphocyte ratio

Supplementary Table S5 : **Baseline parameters according to immune read-outs**
Related to Figure 6
